# Spartan deficiency causes genomic instability and progeroid phenotypes

**DOI:** 10.1038/ncomms6744

**Published:** 2014-12-11

**Authors:** Reeja S. Maskey, Myoung Shin Kim, Darren J. Baker, Bennett Childs, Liviu A. Malureanu, Karthik B. Jeganathan, Yuka Machida, Jan M. van Deursen, Yuichi J. Machida

**Affiliations:** 1 Department of Biochemistry and Molecular Biology, Mayo Clinic, 200 First Street SW, Rochester, Minnesota 55905, USA; 2 Department of Oncology, Mayo Clinic, 200 First Street SW, Rochester, Minnesota 55905, USA; 3 Department of Pediatric and Adolescent Medicine, Mayo Clinic, 200 First Street SW, Rochester, Minnesota 55905, USA; 4 Department of Molecular Pharmacology and Experimental Therapeutics, Mayo Clinic, 200 First Street SW, Rochester, Minnesota 55905, USA

## Abstract

Spartan (also known as DVC1 and C1orf124) is a PCNA-interacting protein implicated in translesion synthesis, a DNA damage tolerance process that allows the DNA replication machinery to replicate past nucleotide lesions. However, the physiological relevance of Spartan has not been established. Here we report that Spartan insufficiency in mice causes chromosomal instability, cellular senescence and early onset of age-related phenotypes. Whereas complete loss of Spartan causes early embryonic lethality, hypomorphic mice with low amounts of Spartan are viable. These mice are growth retarded and develop cataracts, lordokyphosis and cachexia at a young age. Cre-mediated depletion of Spartan from conditional knockout mouse embryonic fibroblasts results in impaired lesion bypass, incomplete DNA replication, formation of micronuclei and chromatin bridges and eventually cell death. These data demonstrate that Spartan plays a key role in maintaining structural and numerical chromosome integrity and suggest a link between Spartan insufficiency and progeria.

## Abstract (web summary)

Spartan/DVC1 is a translesion synthesis regulator with important roles in cellular DNA damage tolerance. Here, the authors report that Spartan is essential for DNA lesion bypass and that Spartan insufficiency in mice causes chromosomal instability, cellular senescence and early onset of age-related phenotypes.

## Graphical abstract

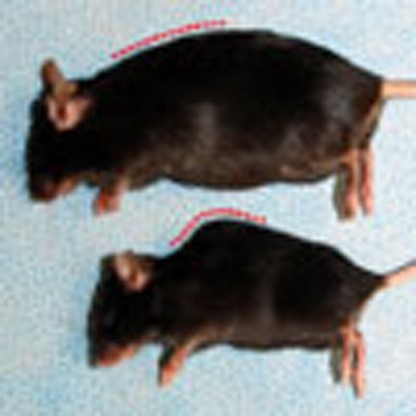

Uninterrupted DNA replication is important for timely duplication of the genome before mitotic entry. However, the replication machinery may stall or slow down on replication stress caused by DNA lesions, limited nucleotide pools and repetitive DNA sequences^1^. Failure to deal with such replication stress could lead to genomic instability, which is associated with cancer and ageing^2^,^3^. In fact, genetic defects in replicative stress response have been implicated in genome instability syndromes that are characterized by increased cancer incidence and/or premature ageing phenotypes^1^. Although replication stress has been commonly linked to cancer susceptibility, accumulating evidence from mouse studies also supports the role of replication stress in premature ageing phenotypes and ageing of stem cells^4^,^5^,^6^.

Genomic DNA lesions are one of the common sources of replication stress. Because high-fidelity replicative polymerases cannot accommodate damaged bases in their active sites, unrepaired DNA lesions pose a risk of replication fork stalling, double-strand DNA breaks and cell death. To avoid such threat to genome stability, cells are equipped with a special replication system called translesion synthesis (TLS), which allows continuous replication across DNA lesions^7^,^8^. During TLS, monoubiquitination of proliferating cell nuclear antigen (PCNA), a sliding clamp for DNA polymerases, induces switching from replicative DNA polymerases to specialized TLS polymerases, thereby allowing bypass of DNA lesions. Although TLS protects replication forks from stalling at DNA lesions, it is potentially mutagenic because it utilizes error-prone DNA polymerases.

Spartan was recently identified as a novel regulator of TLS that guards against TLS-associated mutagenesis^9^,^10^,^11^,^12^,^13^,^14^,^15^. Spartan is recruited to DNA damage sites through the interaction with PCNA via its PIP (PCNA-interacting peptide) motif and the interaction of the zinc-finger domain UBZ4 with ubiquitin on PCNA or other proteins^9^,^10^,^11^,^12^,^13^,^14^. Several groups reported that Spartan promotes monoubiquitination of PCNA^9^,^10^,^14^, thereby facilitating recruitment of TLS polymerases, whereas other groups showed that Spartan recruits an ATP-dependent segregase p97 (also known as valosin-containing protein (VCP)) to the sites of TLS to facilitate dissociation of TLS polymerases^12^,^13^. In addition, our previous study linked Spartan to regulation of the error-prone TLS mechanism involving the DNA polymerase Pol ζ^15^. While these studies clearly implicated Spartan in TLS regulation, whether Spartan is required for TLS is unknown.

In this study, we create *Sprtn*-targeted mouse models and investigate the physiological importance of Spartan. We demonstrate essential roles of Spartan in lesion bypass, completion of DNA replication, genome stability and cell viability. Interestingly, *Sprtn* insufficiency causes senescence and progeria in mice. Our findings establish the critical role of Spartan in the cell cycle and the maintenance of genome integrity and provide the link between Spartan insufficiency and progeria.

## Results

### *Sprtn* knockout causes embryonic lethality in mice

**Figure 1:**
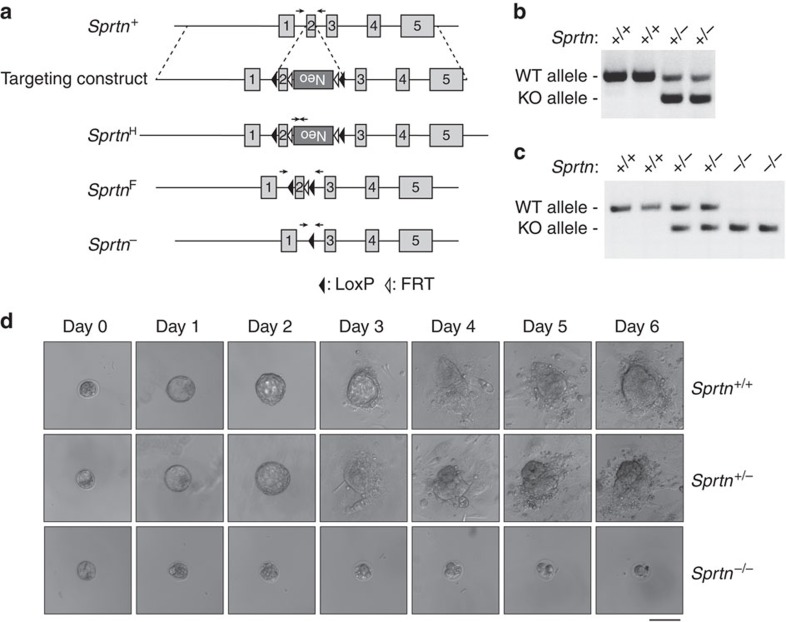
*Sprtn* KO causes embryonic lethality. (**a**) Schematic of the mouse *Sprtn* gene and the targeted alleles. An inverted *Neo* cassette was inserted in the second intron with flanking FLP recognition target (FRT) sequences. LoxP sites were also inserted at the indicated positions. The floxed and KO alleles were created by crossing heterozygote mice with *FLP* and *Cre*-transgenic mice, respectively. Positions of genotyping primers are indicated by arrows. (**b**) PCR-based genotyping (at weaning) of wild-type and *Sprtn* heterozygote mice produced by intercrossing *Sprtn*^+/−^. (**c**) PCR-based genotyping of wild-type, heterozygote and KO blastocysts. (**d**) Blastocysts from *Sprtn*^+/−^ intercrosses were cultured *in vitro* and observed by phase-contrast microscopy on 6 consecutive days. Representative images of *Sprtn*^+/+^, *Sprtn*^+/−^ and *Sprtn*^−/−^ blastocysts are shown. Scale bar, 100 μm.

To explore the physiological relevance of Spartan, we generated a series of mice with graded reduction in Spartan expression using hypomorphic (H) and knockout (KO) *Sprtn* alleles that we generated by gene targeting (Fig. 1a,b). Intercrossing of *Sprtn*^+/−^ mice failed to produce *Sprtn*^−/−^-live offspring and no *Sprtn*^−/−^ embryos were found from embryonic day (E) 7.5 to E13.5 (Table 1), suggesting that *Sprtn* KO in mice causes early embryonic lethality. To examine the effect of *Sprtn* KO on early embryogenesis, we isolated blastocysts at E3.5 and cultured them *in vitro* for 6 days. Genotyping was successful for all the blastocysts collected, and *Sprtn*^−/−^ blastocysts were obtained at normal Mendelian frequency (Fig. 1c; Table 2). However, *Sprtn*^−/−^ blastocysts failed to hatch and enlarge the inner cell mass when cultured *in vitro* (Fig. 1d; Table 2), indicating that death occurred prior at the implantation stage.

### Effects of *Sprtn* KO on cell proliferation and survival

**Figure 2:**
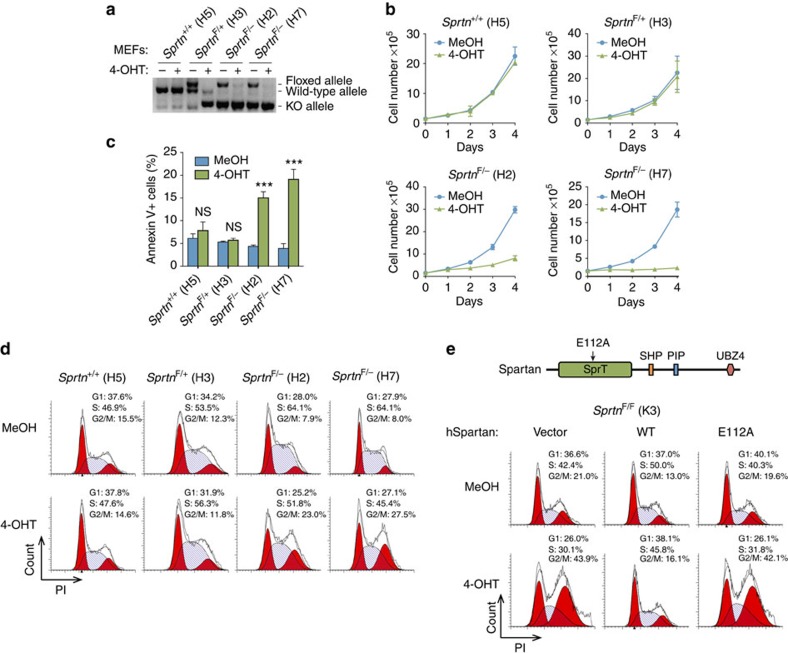
*Sprtn* KO causes impaired cell proliferation and cell death. (**a**) PCR-based genotyping of *Sprtn* alleles in the indicated MEF lines after 48 h treatment with MeOH or 2 μM 4-OHT. (**b**) Proliferation of *Sprtn*-targeted MEFs. Cells treated with MeOH or 4-OHT for 48 h were seeded and cell numbers were counted at the indicated time points. Values are mean±s.d. of three independent experiments. (**c**) Analyses of apoptosis in *Sprtn*-targeted MEFs. Two days after the completion of 48 h treatment with MeOH or 4-OHT, cells were stained with Annexin V and PI and analysed by flow cytometry. Values are mean±s.d. of three independent experiments. NS, not significant; ***P<0.001 (two-tailed unpaired *t*-test). (**d**) Cell cycle profiling of *Sprtn*-targeted MEFs. The indicated MEF lines were treated with MeOH or 4-OHT for 48 h. Two days later, cells were stained with PI and analysed by flow cytometry. (**e**) Cell cycle profiling of *Sprtn*^F/F^; *Cre-ER*^*T2*^ MEFs (K3) expressing wild-type human Spartan or the E112A mutant after 48 h treatment with MeOH or 4-OHT.

To further investigate the role of *Sprtn* at the cellular level, we generated a conditional *Sprtn* KO system in mouse embryonic fibroblasts (MEFs). *Sprtn*^+/+^, *Sprtn*^F/+^ and *Sprtn*^F/−^ MEFs were immortalized and transduced with retroviruses expressing Cre-ER^T2^. Treatment with 4-hydroxytamoxifen (4-OHT) resulted in excision of exon 2, efficiently converting the floxed allele to a KO allele (Fig. 2a). *Sprtn*^F/−^ MEFs, but not *Sprtn*^+/+^ and *Sprtn*^F/+^ MEFs, showed markedly reduced proliferation and increased apoptosis after exposure to 4-OHT (Fig. 2b,c), suggesting that *Sprtn* is essential for cell proliferation and survival.

### Cell cycle effects from the loss of *Sprtn*

To determine whether *Sprtn* KO has any effects on the cell cycle, we examined the cell cycle profile of MEFs with various genotypes by flow cytometry. A large portion of 4-OHT-treated *Sprtn*^F/−^ MEFs accumulated with 4C DNA content after treatment, whereas *Sprtn*^+/+^ and *Sprtn*^F/+^ cells were unaffected (Fig. 2d). To examine S-phase progression of *Sprtn*^−/−^ MEFs more closely, we performed 5-bromodeoxyuridine (BrdU) pulse-chase experiments, in which S-phase cells were pulse labelled with BrdU and chased in BrdU-free media every 2 h (Supplementary Fig. 1a). *Sprtn*^−/−^ MEFs initially progressed through S phase at a similar rate to wild-type cells, but subsequently arrested with 4C DNA content at high rates (Supplementary Fig. 1b). These results suggest that *Sprtn*^−/−^ cells exhibit normal S-phase progression, but accumulate in late S or G2/M phases. A relatively small proportion of *Sprtn*^−/−^ MEFs were positive for p-H3^Ser10^, a well-established mitotic marker^16^,^17^, indicating that *Sprtn*^−/−^ cells accumulate before entry into mitosis (Supplementary Fig. 1c). Collectively, these results suggest that Spartan is essential for cell proliferation and faithful progression through late S and G2 phases.

The Spartan protein possesses multiple domains including SprT (a zinc metalloprotease-like domain), a SHP box (a p97-interacting motif), a PIP box (a PCNA-interacting motif) and UBZ4 (a ubiquitin-binding domain) (Fig. 2e, top). We asked whether these domains are necessary for the function of Spartan in the cell cycle. Wild-type human Spartan, which was expressed at the similar level to endogenous Spartan in a human fibroblast cell line (Supplementary Fig. 2a), suppressed accumulation of cells in late S or G2 phase following loss of *Sprtn* in MEFs (Fig. 2e). On the other hand, Spartan^E112A^, a mutant with a substitution in the putative active site of the metalloprotease-like domain SprT^15^, failed to rescue the cell cycle defect in *Sprtn*^−/−^ MEFs despite the similar expression level to wild-type Spartan (Fig. 2e; Supplementary Fig. 2a). In contrast, the ectopic expression of human Spartan^PIP*^ and Spartan^UBZ*^, which exhibit diminished interactions with PCNA and ubiquitin, respectively^11^, efficiently suppressed the cell cycle defect in *Sprtn*^−/−^ MEFs (Supplementary Fig. 2b,d). Similarly, mouse Spartan^SHP*^, which harbours the mutations that diminish interaction with p97 (ref. 13), also suppressed the cell cycle defect in *Sprtn*^−/−^ MEFs as efficiently as wild-type Spartan (Supplementary Fig. 2c,e). Altogether, these results suggest that the putative active site of the SprT domain is essential for the cell cycle function of Spartan, while the PIP, UBZ and SHP domains are dispensable.

### Loss of Spartan causes DNA damage and checkpoint activation

**Figure 3:**
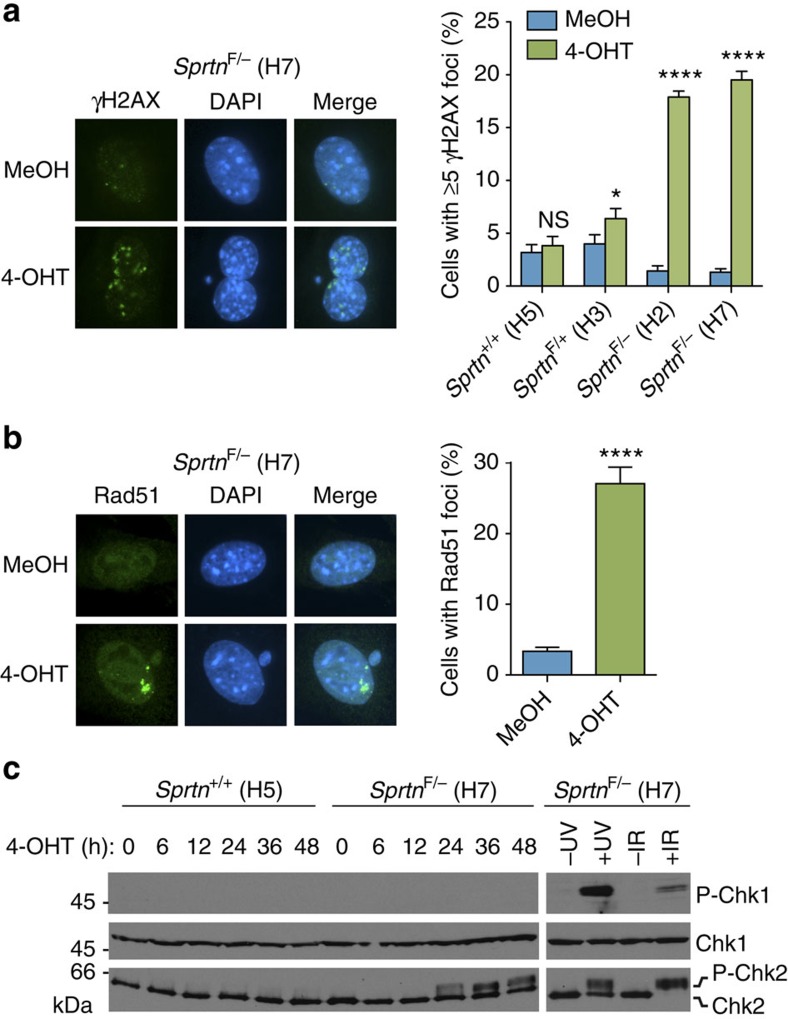
*Sprtn* KO causes DNA damage and checkpoint activation. (**a**) γH2AX focus formation. The indicated MEFs treated with MeOH or 4-OHT for 48 h were stained with anti-γH2AX. At least 300 cells were scored for γH2AX foci and percentages of cells with 5 or more foci are shown. Values are mean±s.d. of three independent experiments. NS, not significant; *P<0.05; ****P<0.0001 (two-tailed unpaired *t*-test). (**b**) Rad51 focus formation. The indicated MEFs were stained with anti-Rad51 after 48 h treatment with MeOH or 4-OHT. At least 300 cells were scored for Rad51 foci. Experiments were performed in triplicate and mean±s.d. is shown. ****P<0.0001 (two-tailed unpaired *t*-test). (**c**) Western blot analyses of phospho-Chk1 and Chk2. The indicated MEFs were treated with 4-OHT and harvested at various time points. *Sprtn*^F/−^ (H7) cells treated with ultraviolet (40 J m^−2^) or ionizing radiation (10 Gy) are shown as positive controls for checkpoint kinases activation. Chk1 is used as a loading control. P-Chk1, phospho-Chk1 (Ser345); P-Chk2, phospho-Chk2. Uncropped blots are shown in Supplementary Fig. 7.

To determine the cause of the cell cycle defect in *Sprtn*^−/−^ MEFs, we asked whether Spartan deficiency engages the DNA damage response pathway. γH2AX foci, a marker of DNA damage^18^, markedly increased in *Sprtn*^F/−^ MEFs after 4-OHT treatments, but not in *Sprtn*^+/+^ and *Sprtn*^F/+^ MEFs (Fig. 3a). Consistent with increased DNA damage, *Sprtn*^−/−^ MEFs showed high rates of double-strand DNA breaks as measured by immunostaining of the DNA recombination protein Rad51 (Fig. 3b) and profound activation of the checkpoint kinase Chk2 (Fig. 3c). Thus, loss of *Sprtn* in MEFs seemingly induces DNA damage, most likely DNA breaks, leading to activation of the DNA damage checkpoint.

### Depletion of Spartan causes genomic instability

**Figure 4:**
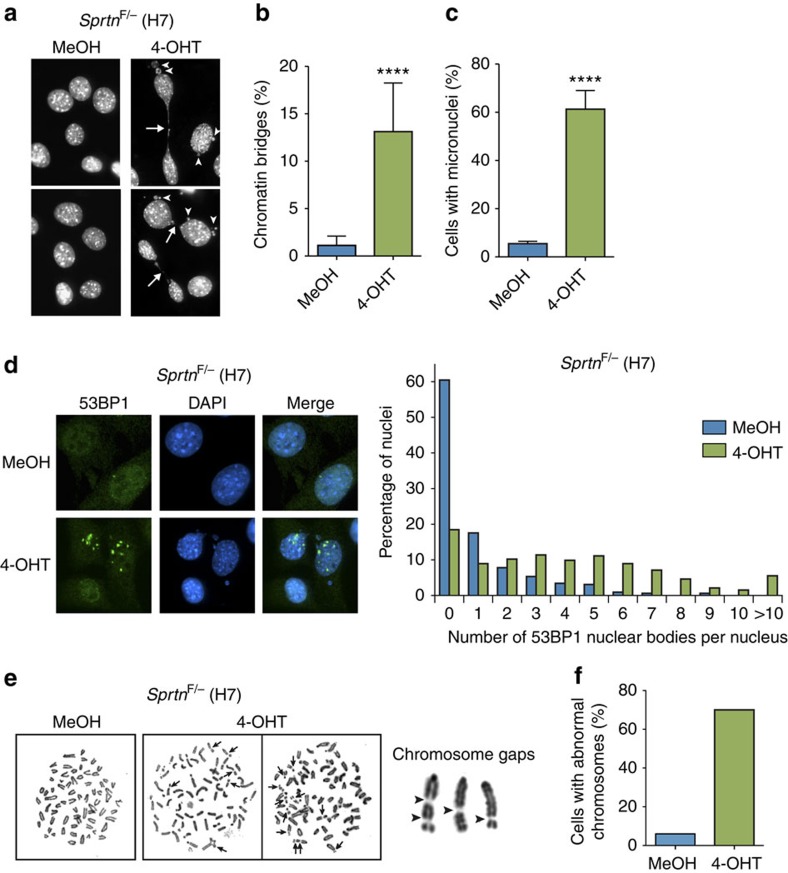
Genome instability in *Sprtn* KO MEFs. (**a**) Images showing chromatin bridges (arrows) and micronuclei (arrowheads) in *Sprtn*^F/−^ MEFs treated with MeOH or 4-OHT for 48 h. DNA was visualized by DAPI staining. (**b**) Quantitation of chromatin bridges. Experiments were performed as in **a**. At least 300 cells were scored for chromatin bridges and percentages of positive cells are shown. Three independent experiments were performed and mean±s.d. is shown. ****P<0.0001 (two-tailed unpaired *t*-test). (**c**) Quantitation of micronuclei-containing cells. Experiments were performed as in **a**. ****P<0.0001 (two-tailed unpaired *t*-test). (**d**) Formation of 53BP1 nuclear bodies. *Sprtn*^F/−^ MEFs were treated with MeOH or 4-OHT for 48 h. At least 300 cells were scored for 53BP1 nuclear bodies and percentages of nuclei with different number of 53BP1 nuclear bodies per nucleus are shown. (**e**) Chromosomal abnormalities in *Sprtn* KO cells. *Sprtn*^F/−^ MEFs were treated with MeOH or 4-OHT for 48 h. Twelve hours after completion of the treatments, mitotic spreads were prepared and examined by microscopy. Representative pictures of mitotic spreads are shown in the left panel. Arrows indicate some of the chromosome abnormalities. In the right panel, representative images of chromosome gaps in *Sprtn* KO MEFs are indicated by arrowheads. (**f**) Quantitation of abnormal chromosomes. Cells harbouring chromosomal abnormalities were scored in 50 mitotic spreads of *Sprtn*^F/−^ MEFs treated with MeOH or 4-OHT.

The elevated levels of DNA damage accompanied by checkpoint activation in *Sprtn*^−/−^ MEFs prompted us to assess whether loss of Spartan induces genomic instability. Microscopic analysis of 4',6-diamidino-2-phenylindole (DAPI)-stained MEFs revealed that a substantial fraction of mitotic *Sprtn*^−/−^ MEFs exhibited chromatin bridges and micronuclei, structures that have been associated with unresolved replication intermediates and unrepaired DNA breaks^19^ (Fig. 4a–c). In addition, these MEFs had increased numbers of 53BP1 nuclear bodies that signify incomplete DNA replication^20^,^21^ (Fig. 4d). These data raise the possibility that the genome of *Sprtn*-deficient cells is subject to under-replication. Consistent with this interpretation, the vast majority of chromosomal abnormalities observed in the mitotic spreads of *Sprtn*^−/−^ MEFs were chromosome gaps, which might represent under-replicated regions (Fig. 4e,f). In summary, these results suggest that Spartan is crucial for maintaining genome integrity.

### Lesion bypass defects in *Sprtn* ^−/−^ cells

**Figure 5:**
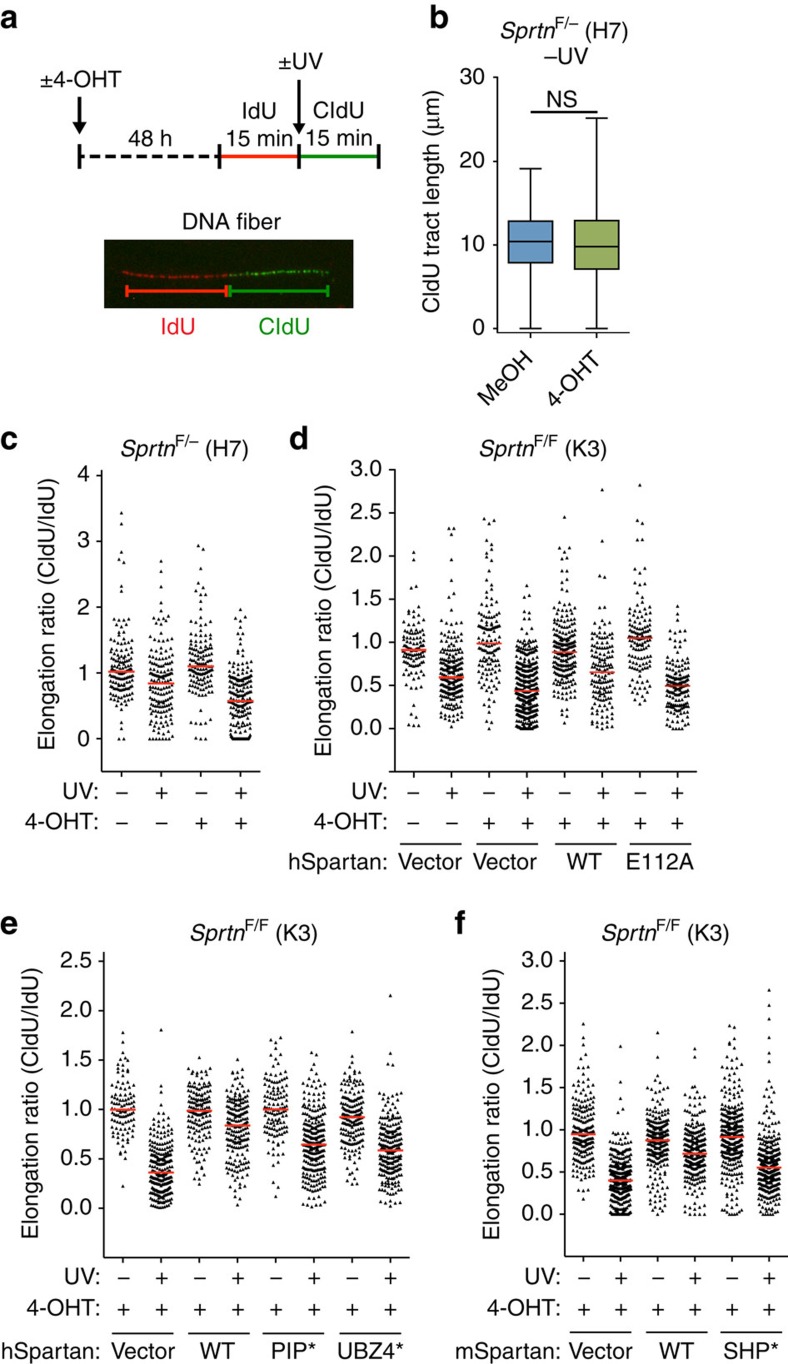
Effects of *Sprtn* KO on DNA replication forks. (**a**) Schematic representation of DNA fiber assays. MEFs were treated with MeOH or 4-OHT for 48 h and sequentially labelled with IdU and CldU to mark ongoing replication. A picture of a representative replication track is shown. At least 100 fibres were scored for each sample in all of the DNA fiber experiments. (**b**) A box plot showing distribution of the lengths of CldU tracts in *Sprtn*^F/−^ MEFs treated with MeOH or 4-OHT for 48 h. NS, not significant (*P*=0.829, two-tailed unpaired *t*-test). (**c**) Effect of ultraviolet irradiation on replication forks. DNA fiber assays were performed with *Sprtn*^F/−^ MEFs with or without ultraviolet irradiation (40 J m^−2^) between IdU and CldU labelling. Distribution of replication forks at different CldU/IdU ratios is shown. (**d**–**f**) Effect of ultraviolet irradiation on replication forks. Experiments were performed as in **c** using *Sprtn*^F/F^; *Cre-ER*^*T2*^ MEFs (K3) expressing wild-type human Spartan or the E112A mutant (**d**), wild-type human Spartan, the PIP* or the UBZ* mutant (**e**), and wild-type mouse Spartan or the SHP* mutant (**f**). Horizontal red lines in **c**–**f** indicate median values.

To examine the effect of *Sprtn* inactivation on DNA replication more directly, we measured movement of replication forks in *Sprtn*^−/−^ MEFs using DNA fiber assays, in which replication tracts were sequentially labelled with the thymidine analogues IdU and CldU (Fig. 5a). Lengths of CldU-labelled DNA in *Sprtn*^−/−^ MEFs were not significantly different from those of *Sprtn*^+/+^ MEFs (Fig. 5b), suggesting that the majority of replication forks are not affected by Spartan loss, an observation consistent with the normal S-phase progression in *Sprtn*^−/−^ cells (Supplementary Fig. 1b).

Given that *Sprtn*^−/−^ cells have mostly intact replication forks but still accumulate in late S/G2 phases accompanied by under-replication phenotypes, we hypothesized that Spartan might be important for DNA replication under stress, for example, imposed by endogenously created DNA lesions. Previous reports have indeed linked Spartan’s function to TLS^9^,^10^,^11^,^12^,^13^,^14^,^15^, although whether Spartan is necessary for the bypass of DNA lesions during DNA replication has not been established. We therefore tested the requirement of Spartan for lesion bypass during DNA replication. DNA fiber assays were performed with ultraviolet light exposure, which introduces lesions acted on by TLS, between IdU and CldU labelling (Fig. 5a). Lesion bypass efficiency is expressed as elongation ratios (CldU/IdU), which would be reduced with lesion bypass defects. Strikingly, *Sprtn* KO in MEFs caused reduced fork progression at ultraviolet-induced lesions (Fig. 5c), suggesting that Spartan is important for ultraviolet-induced lesion bypass. We also examined whether Spartan plays a role in the stability of stalled forks by measuring replication fork restart after hydroxyurea (HU) treatments (Supplementary Fig. 3a). *Sprtn* KO did not increase the amount of collapsed forks on HU treatments (Supplementary Fig. 3b), suggesting that Spartan is not necessary for replication restart after fork stalling.

Next we assessed the requirement of the Spartan domains for the bypass of ultraviolet-induced lesions by re-expressing Spartan mutants in conditional *Sprtn* KO MEFs. The lesion bypass defect in *Sprtn*^−/−^ cells was rescued by the introduction of wild-type human Spartan, but not the mutant that harbours the E112A mutation in the putative active site (Fig. 5d), suggesting that the role of Spartan in lesion bypass requires an intact SprT domain. On the other hand, the PIP*, UBZ4* or SHP* mutants of Spartan exhibited partially diminished abilities to rescue the lesion bypass defects in *Sprtn*^−/−^ MEFs (Fig. 5e,f). Taken together, these results suggest that Spartan is dispensable for the replication of the bulk of the genome, but is crucial for replication past DNA damage. This function of Spartan in lesion bypass depends on the putative active site of the SprT domain, but only partially on the PIP, UBZ4 and SHP domains.

To obtain more insight into the role of Spartan in lesion bypass, we examined the effect of *Sprtn* KO on PCNA ubiquitination in response to ultraviolet irradiation and dissociation of Pol η from ultraviolet-induced DNA damage sites, the two processes that have been reported to be impaired in Spartan-depleted human cells^9^,^10^,^12^,^13^,^14^. PCNA ubiquitination after ultraviolet irradiation was largely intact in *Sprtn*^−/−^ cells (Supplementary Fig. 3c), suggesting that Spartan is not absolutely necessary for ultraviolet-induced PCNA ubiquitination. On the other hand, in agreement with previous reports^12^,^13^, we observed prolonged retention of enhanced green fluorescent protein (EGFP)-Pol η foci after ultraviolet irradiation in *Sprtn*^−/−^ cells (Supplementary Fig. 3d), although it remains to be determined whether this is due to a failure to extract Pol η from DNA damage sites as proposed previously^12^,^13^ or it reflects the lesion bypass defects we report in this study (Fig. 5c).

### Growth defects and genome instability in *Sprtn* ^H/−^ cells

Finally, we examined the effect of *Sprtn* insufficiency by producing *Sprtn* hypomorphic mice. The targeted *Sprtn*^Neo^ allele is hypomorphic (hereafter referred to as *Sprtn*^H^), producing wild-type mRNAs at reduced levels due to the cryptic exon in the Neo cassette^22^ (Supplementary Fig. 4a). In contrast to *Sprtn*^−/−^ MEFs, *Sprtn*^H/−^ MEFs, which contain one KO and one hypomorphic allele that produce reduced amounts of wild-type Spartan, were viable, but proliferated at reduced rates (Supplementary Fig. 4b–d). Furthermore, *Sprtn*^H/−^ MEFs had increased rates of aneuploidy and micronuclei (Supplementary Fig. 4e,f; Supplementary Table 1), indicating that, in addition to structural chromosome damage, Spartan insufficiency leads to numerical chromosome instability.

### Growth defects and genomic instability in *Sprtn* ^H/H^ mice

**Figure 6:**
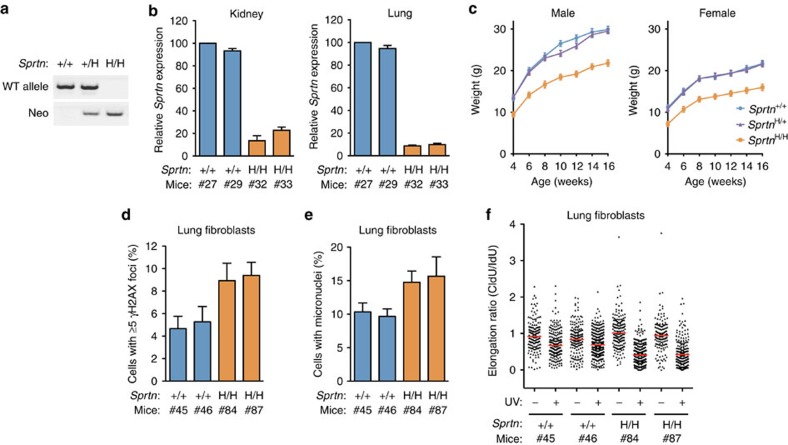
Characterization of *Sprtn* hypomorphic cells and mice. (**a**) PCR-based genotyping of *Sprtn*^+/+^, *Sprtn*^+/H^ and *Sprtn*^H/H^ mice at weaning. (**b**) Quantitative PCR analyses of *Sprtn* mRNA levels in *Sprtn*^+/+^ and *Sprtn*^H/H^ mice. *Sprtn* mRNA levels in the kidneys (left) and lungs (right) of two mice per genotype were measured three times by quantitative reverse transcription PCR and mean±s.d. is shown. Values were normalized to *Gapdh* and shown relative to *Sprtn*^+/+^ mouse #27. (**c**) Growth curves of *Sprtn*^+/+^, *Sprtn*^+/H^ and *Sprtn*^H/H^ mice (*n*=5, mean±s.d.) of 4–16 weeks. (**d**,**e**) Quantitation of cells containing five or more γH2AX foci (**d**) and micronuclei (**e**) in the indicated *Sprtn*^+/+^ and *Sprtn*^H/H^ primary lung fibroblasts. At least 300 cells were scored in each experiment and percentages of positive cells are shown. Values are mean±s.d. of three independent experiments. For **d**,**e**, statistical significance is *P*<0.001 (H/H group versus +/+ group), two-tailed unpaired *t*-test. (**f**) Effect of ultraviolet irradiation on replication forks. DNA fiber assays were performed with *Sprtn*^+/+^ and *Sprtn*^H/H^ primary lung fibroblasts with or without ultraviolet irradiation (40 J m^−2^) between IdU and CldU labelling. Distribution of replication forks at different CldU/IdU ratios is shown. A horizontal red line indicates median value.

Although no viable *Sprtn*^H/−^ offspring were found in crossing of *Sprtn*^+/−^ and *Sprtn*^+/H^ mice, *Sprtn*^H/H^ mice were born at near Mendelian frequency from *Sprtn*^+/H^ breeding pairs (*Sprtn*^+/+^, *Sprtn*^+/H^ and *Sprtn*^H/H^ at 26.6, 53.3 and 20.1%, respectively), despite substantially reduced *Sprtn* expression (Fig. 6a,b). At weaning, *Sprtn*^H/H^ mice appeared healthy but were overtly smaller than control littermates. Dwarfism was observed in both sexes and persisted as *Sprtn*^H/H^ mice developed to adulthood (Fig. 6c). To investigate in more detail the effects of *Sprtn* insufficiency in *Sprtn*^H/H^ mice, we isolated primary lung fibroblasts and prostate epithelial cells from *Sprtn*^+/+^ and *Sprtn*^H/H^ mice. *Sprtn* expression was reduced to less than 10% in the *Sprtn*^H/H^ cells (Supplementary Fig. 5a,b). *Sprtn*^H/H^ lung fibroblasts showed elevated structural and numerical chromosomal instabilities, as evidenced by increased rates of γH2AX foci, aneuploidy and micronuclei (Fig. 6d,e; Table 3). In addition, *Sprtn*^H/H^ lung fibroblasts displayed defects in fork progression at ultraviolet-induced lesion, which was also confirmed in *Sprtn*^H/H^ prostate epithelial cells (Fig. 6f; Supplementary Fig. 5c). Taken together, these results indicate that cells from *Sprtn* hypomorphic mice exhibit genome instability and lesion bypass defects.

### Reduced expression of *Sprtn* causes accelerated ageing in mice

**Figure 7:**
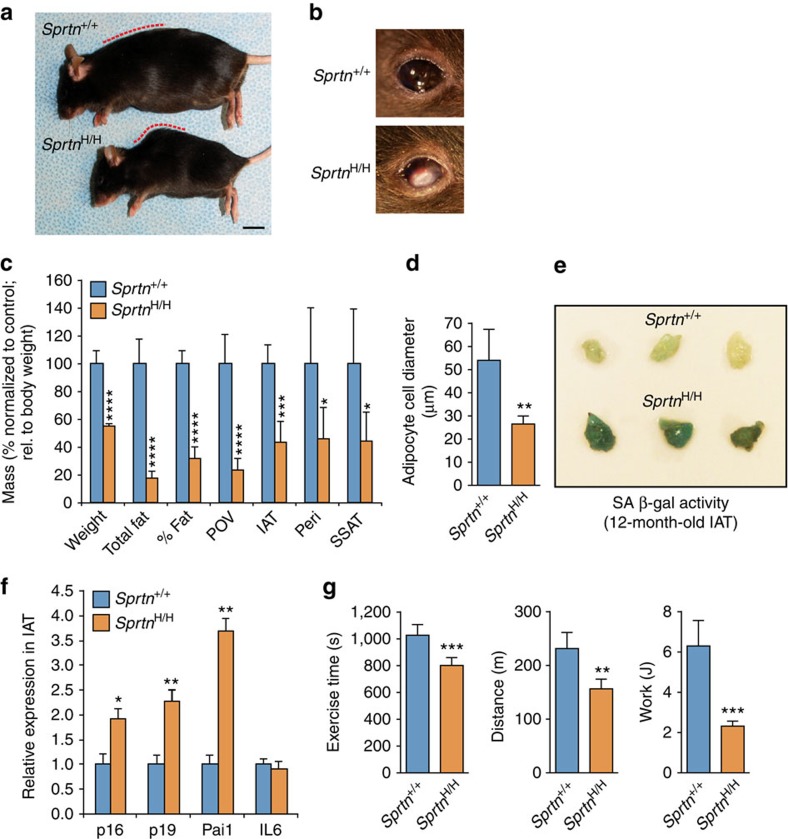
Premature ageing phenotypes in *Sprtn*^H/H^ mice. (**a**) Representative images of 12-month-old *Sprtn*^+/+^ and *Sprtn*^H/H^ female mice. Note lordokyphosis in *Sprtn*^H/H^ indicated by the dotted red line. Scale bar, 1 cm. (**b**) Representative images of the eyes of *Sprtn*^+/+^ and *Sprtn*^H/H^ female mice. Note a cataract in *Sprtn*^H/H^. (**c**) Body weight, fat mass, body fat percentage and adipose depot weights of 12-month-old *Sprtn*^+/+^ and *Sprtn*^H/H^ female mice (*n*=5, mean±s.d.). Values are normalized to the average of the *Sprtn*^+/+^ mice. Because of the smaller body size of the *Sprtn*^H/H^ mice, adipose depot measurements were first calculated relative to body weight. (**d**) Fat cell diameter measurements (*n*=5, mean±s.d.). (**e**) IAT of 12-month-old mice stained for SA-β-gal activity. (**f**) Relative expression of senescence markers in 12-month-old IAT (*n*=5, mean±s.d.). Values were normalized to *Gapdh* and are relative to *Sprtn*^+/+^ IAT. (**g**) Treadmill exercise ability of 12-month-old *Sprtn*^+/+^ and *Sprtn*^H/H^ female mice. Exercise time, distance travelled and workload performed are shown (*n*=5, mean±s.d.). For all panels, statistical significance is as follows: *P<0.05, **P<0.01, ***P<0.001, ****P<0.0001 (two-tailed unpaired *t*-test).

As young adults, *Sprtn*^H/H^ mice exhibited no overt phenotypes other than dwarfism. However, by 12 months of age, all *Sprtn*^H/H^ mice (seven out of seven mice) had developed lordokyphosis (concavity in the curvature of the lumbar and cervical spine), cataracts and cachexia (Fig. 7a,b), three phenotypes often seen in mouse models of progeria^23^,^24^,^25^. The total fat mass and percentage of body fat of these mice were dramatically reduced (Fig. 7c). In-depth analysis of individual fat depots revealed that the relative weights of the paraovarian, inguinal adipose tissue (IAT), perirenal and subscapular adipose tissue were all significantly reduced in size (Fig. 7c). Consistent with fat tissue atrophy, fat cells in *Sprtn*^H/H^ IAT were significantly smaller in size (Fig. 7d). Furthermore, IAT stained highly positive for senescence-associated-β-galactosidase (SA-β-GAL) and showed increased levels of key senescence markers, including p16^Ink4a^, p19^Arf^ and PAI-1 (Fig. 7e,f). These data suggest that increased accumulation of cellular senescence in fat causes dysfunction and atrophy of this tissue. Lordokyphosis in progeroid mouse models is often associated with osteoporosis and/or muscle wasting^24^,^26^. However, neither disorder seemed to apply to *Sprtn*^H/H^ mice as their bone mineral content and density (Supplementary Fig. 6a,b) and gastrocnemius, abdominal and paraspinal muscle fibre diameters (Supplementary Fig. 6c–e) were normal. Consistent with segmental progeria, *Sprtn*^H/H^ mice showed impaired exercise ability as measured by the use of a treadmill: the duration of exercise, the distance travelled and the overall amount of work performed were all reduced at the age of 12 months (Fig. 7g). Taken together, these data suggest that the reduced expression of *Sprtn* leads to development of various progeroid phenotypes, including dwarfism, cataracts, lordokyphosis, fat tissue dysfunction and accumulation of senescent cells.

## Discussion

In this study, we investigate the physiological roles of Spartan using a series of mice with graded reduction in *Sprtn* expression. Our data uncover a striking requirement of Spartan in cell survival and mouse development. Specifically, *Sprtn* KO causes early embryonic lethality in mice, while conditional KO in MEFs leads to incomplete DNA replication and cell death. In contrast, *Sprtn* hypomorphic mice are viable but develop premature ageing phenotypes accompanied by elevated levels of DNA damage and genome instability. Extending previous studies that implicated Spartan in TLS, we demonstrate that lesion bypass is impaired in *Sprtn* KO and hypomorphic cells when assayed with external DNA damage. Taken together, our data best fit a model whereby Spartan is essential for DNA replication under stress, which can be imposed by DNA lesions or difficult-to-replicate sequences such as repetitive DNA elements. This model is also consistent with previous studies that have linked replication stress with age-related phenotypes in mice^4^,^5^,^6^. In summary, our findings reveal the fundamental role of Spartan in the cell cycle and genome stability and support the link between deficiency in replication stress response and progeria.

To our knowledge, the current study is the first to demonstrate the requirement of Spartan in lesion bypass. This suggests that Spartan plays a more fundamental function than just suppressing damage-induced mutations. Our experiments using a various Spartan mutants provide mechanistic insight into the role of Spartan in lesion bypass. The results indicate that bypass of ultraviolet-induced lesions is dependent on the SprT domain of Spartan but only partially on the SHP, PIP and UBZ4 domains (Fig. 5d–f). The dependence on the SprT domain is consistent with our previous observation that suppression of ultraviolet-induced mutagenesis by Spartan required an intact SprT domain^15^. On the other hand, the partial requirement of the PIP and UBZ4 domains indicates that recruitment of Spartan to ubiquitinated PCNA is important, but not absolutely necessary for Spartan’s function in lesion bypass. This is consistent with the report that PCNA ubiquitination is important but not absolutely required for TLS^27^. Although not absolutely required, the SHP box of Spartan clearly has an important role in lesion bypass. Previous reports suggested a model in which the Spartan-p97 interaction facilitates p97-mediated removal or degradation of Pol η from DNA damage sites. Consistent with this model, *Sprtn*^−/−^ MEFs showed prolonged retention of ultraviolet-induced Pol η foci (Supplementary Fig. 3d). Our findings that both the SprT domain and the SHP box are involved in lesion bypass raise an intriguing possibility that p97 and the SprT domain of Spartan cooperate in polymerase switching at the sites of TLS.

Although it is not clear why *Sprtn* KO cells die, it is tempting to speculate that failure to execute TLS is a major contributing factor given that the catalytic subunit of the TLS polymerase Pol ζ has been shown to be essential in mice^28^,^29^,^30^,^31^,^32^,^33^. Consistently, our previous study implicated Spartan in regulation of Pol ζ^15^. Therefore, Spartan might be required for efficient TLS in response to stalled replication at endogenous DNA damage sites, thereby allowing cells to complete S phase. In addition, given that TLS polymerases are important for efficient replication and stability of difficult-to-replicate sequences^34^,^35^,^36^,^37^, it is possible that Spartan-deficient cells exhibit cell cycle defects and genome instability due to replication failure at the sites of inherent replication stress such as difficult-to-replicate regions. Alternatively, given the previous observation that Spartan interacts with the DNA polymerase δ subunit^10^,^15^, it is possible that Spartan has a more general role in DNA replication unrelated to the TLS pathway. In this case, Spartan might be dispensable for replication of the majority of the genome, with only certain regions requiring Spartan function. Our experiments found that distinguishing these two possibilities might be challenging, because PIP and UBZ4, two TLS-linked domains of Spartan, were not only dispensable for cell viability (Supplementary Fig. 2d), but also not completely required for the function of Spartan in lesion bypass (Fig. 5e). In contrast, we found that the SprT is necessary for both lesion bypass and cell viability (Fig. 5d). Therefore, functional studies of the putative zinc metalloprotease domain in TLS or other processes will clarify the cause of incomplete DNA replication and cell death following *Sprtn* KO.

We find that Spartan deficiency leads to both structural and numerical chromosome instabilities, both of which have been associated with cancer predisposition. *Sprtn*^H/H^ mice sacrificed at 1 year of age were thoroughly screened for the presence of tumours, but none were detected. On the other hand, with 100% penetrance, these mice presented multiple age-related and progeroid phenotypes, suggesting that the genomic stress resulting from Spartan insufficiency predominantly engages signalling pathways that have been associated with cell fate decisions that negatively impact tissue homeostasis and repair, such as the p53 and the p16^Ink4a^ tumour suppressor pathways^38^. Consistent with this, we observed a profound accumulation of senescent cells in fat tissue of Spartan-insufficient mice, as well as increased levels of p16^Ink4a^. Several genome maintenance syndromes have exclusively been associated with progeria, including Cockayne syndrome, trichothiodystrophy, Rothmund-Thomson and dyskeratosis congenital, while others, including Fanconi anaemia, xeroderma pigmentosum, ataxia telangiectasia, Nijmegen breakage syndrome, Bloom syndrome and Werner syndrome, cause both cancer and progeroid phenotypes^39^. Our data suggest that Spartan insufficiency primarily causes progeria; however, it will be important to screen a larger cohort of *Sprtn*^H/H^ mice for tumour formation in future studies. While this manuscript was under review, Lessel *et al*.^40^ reported biallelic germline mutations in *Sprtn* in human patients with a new segmental progeroid syndrome. Accelerated ageing in *Sprtn* hypomorphic mice in our study is consistent with the progeria in the human patients, and our study provides a firm link between Spartan insufficiency and premature ageing.

In conclusion, the current study provides the evidence that Spartan is essential for the normal cell cycle and cell viability. Our data strongly suggest that Spartan deficiency causes cell cycle defects through accumulation of incompletely replicated genomic regions. Further studies on the potential enzyme activity of the SprT domain will reveal the full function of Spartan and provide insight into the mechanism by which insufficiency of Spartan leads to genome instability and progeria.

## Methods

### Generation of *Sprtn*-targeted mice

The *Sprtn*-targeting vector was constructed in modified pNTKV1901-frt/loxP^22^ with a new MfeI site for left homology arm cloning. Mouse *Sprtn* genomic sequences were amplified by PCR from a BAC clone (RPCI-23 bMQ384-H14) using following primers. Left homology arm forward, 5′- GCATCAGATATCTAAAGGCTTCCTTCCACATGCTAC -3′; left homology arm reverse, 5′- GCATCACAATTGATCTCCGAGTTCCAGGCCAGC -3′; exon 2 forward, 5′- GCATCAAAGCTTGAATTCCCTCCTGCATCTGCCTTCCACG -3′; exon 2 reverse, 5′- GCATCAGGTACCGTATGGGCTGCTATCAAGAACC -3′; right homology arm forward, 5′- GCATCACCGCGGAAGCTTTGGCTTCAACCACAAGATCCTC -3′; right homology arm reverse, 5′- GCATCAGTCGACTCAGGGACTCCAGATAAGACTAC -3′. After electroporation into 129Sv/E mouse embryonic stem cells and selection under G418, correctly targeted clones were injected into blastocysts and resulting chimeric mice were used to generate *Sprtn*^Neo/+^ mice. *Sprtn*^+/−^ mice were generated by crossing *Sprtn*^Neo/+^ mice and *Cre*-transgenic mice. A conditional KO allele (F allele), in which exon 2 is floxed, was created by crossing *Sprtn*^Neo/+^ mice with *FLP*-transgenic mice. *Sprtn*^F/+^ mice were crossed with *Cre-ER*^*T2*^-transgenic mice (B6.129-*Gt(ROSA)26Sor*^*tm1(cre/ERT2)Tyj*^/J)^41^ to generate *Sprtn*^F/+^; *Cre-ER*^*T2*^ mice. *Sprtn*^H/H^ mice were generated by intercrossing *Sprtn*^Neo/+^ mice. All mice were maintained on a mixed 129Sv/E × C57BL/6 genetic background. Genotyping was performed by PCR analyses of tail DNA using three primers: wild-type-specific forward (5′- GTGCTGGGATCTGCACCTAT -3′), KO-specific forward (5′- CCATCAGGGACGTTTTCTTG -3′) and common reverse (5′- TGCACAGCTGTAAACCCTTG -3′). To detect the *Neo* (H) allele, Neo forward (5′- TCCCAAGTGCTGGGATTAAG -3′) and Neo reverse (5′- TCGCCTTCTTGACGAGTTCT -3′) primers were used. PCR conditions were 35 cycles of 94 °C for 30 s, 60 °C for 30 s and 72 °C for 1 min. The PCR products are 425 bp (base pair) for the wild-type allele, 278 bp for the KO allele, 527 bp for the floxed allele and 442 bp for the *Neo* (H) allele. All of the animal procedures were approved by Mayo Clinic Institutional Animal Care and Use Committee.

### *In vitro* culture of mouse blastocysts

All blastocysts were generated by natural mating of 2- to 3-month old *Sprtn*^+/−^ male and *Sprtn*^+/−^ female mice. The morning of the day on which a vaginal plug was detected was designated as day E0.5. Blastocysts were collected on E3.5 by flushing the uteri with M2 medium (Sigma, #M7167) and cultured on 35-mm glass-bottom dishes (FluoroDish FD35, World Precision Instruments) in G-1^TM^ v5 PLUS medium (Vitrolife, #10128) under paraffin oil (Vitrolife, #10029). Each blastocyst was photographed daily using a Zeiss Axio Observer Z1 system with CO_2_ Module S (5% CO_2_), TempModule S (37 °C), Heating Unit XL S (37 °C), A-Pln 10X/0.25 Ph1 objective, AxioCam MRm camera and AxioVision 4.8 software. Images were exported as jpeg files, subsequently cropped and adjusted for brightness and contrast. At day 6, the blastocyst-outgrowing cells or the undeveloped blastocysts were collected using a microneedle and suspended in 50 μl lysis buffer (60 mM Tris–HCl, pH 9.0, 15 mM [NH_4_]_2_SO_4_, 2 mM MgCl_2_, 0.5% Tween-20 (v/v) and 250 μg ml^−1^ proteinase K)^42^. For PCR-based genotyping, 3–10 μl from each sample was used in 25 μl reactions. Genotyping was performed in duplicate and was successful for all of the blastocysts collected.

### Analysis of ageing-associated phenotypes

Body composition was determined using an EchoMRI-100TM QNMR instrument (Echo Medical Systems, Houston, Texas) in 12-month-old female mice. Total body fat and lean mass were measured. Total body fat percentage is equal to fat mass divided by body mass. Bone mineral density and bone mineral content were determined with dual-energy X-ray absorptiometry scanning using a Lunar PIXImus densitometer. Forelimb strength (N) was determined using a grip strength meter from Columbus Instruments. Three consecutive days of acclimation occurred before testing. Treadmill exercise tests were performed as previously described^43^. Briefly, the mice were put on the treadmill that was programmed at 5 m min^−1^ of speed for 2 min, which after 2 min was increased to 8 m min^−1^. The speed of the treadmill was then increased every 2 min at the rate of 2 m min^−1^. The exercise time and distance travelled were determined at the point when the mice were unable to move along the treadmill. Work (J) done was determined by: mass (kg) × *g* (9.8 m s^−2^) × distance (m) × sin(*θ*) (where, *θ*=5°). Individual adipose depots and gastrocnemius muscle were isolated and weighed on sacrifice. All weights were calculated relative to total body weight. Adipose depot measurements were normalized to the average of the five control animals. Formalin-fixed, paraffin-embedded inguinal adipose (IAT) cell and skeletal muscle fibre diameter measurements were performed using a calibrated computer program (Olympus Microsuite Five)^43^. Fifty fibres per sample were measured. To determine SA-β-gal activity, IAT was fixed with fixative solution containing 2% formaldehyde and 0.2% glutaraldehyde for 12 min at room temperature, followed by 12 h incubation with β-galactosidase staining solution containing 1 mg ml^−1^ X-gal at 37 °C.

### Plasmids and viral infection

Complementary DNA fragments encoding wild-type, the E112A mutant, the YFAA mutant (PIP*) and the D473A mutant (UBZ4*) of human Spartan^11^,^15^ were subcloned into a retrovirus vector pMSCV-puro. Wild-type mouse Spartan and Spartan^SHP*^ (harbouring F254A and L261A mutations) were cloned into pMSCV-puro with an amino-terminal 3xFlag tag. Full-length Pol η cDNA was subcloned from pEGFP-C-pol η (a gift from Chikahide Masutani and Fumio Hanaoka) into a lentiviral expression vector pLVX6-IRES-Neo (N-terminal EGFP tag). Retroviruses and lentiviruses were packaged by cotransfecting viral and packaging plasmids in a human embryonic kidney cell line 293T (American Type Culture Collection, CRL-11268). Cells were infected with viral vectors in the presence of 2 μg ml^−1^ polybrene and selected with 3 μg ml^−1^ puromycin or 400 μg ml^−1^ G418.

### Cell culture

MEFs were cultured in Dulbecco’s modified Eagle Medium (DMEM) supplemented with 10% fetal bovine serum (FBS). Primary *Sprtn*^H/−^ MEFs were isolated from E13.5 embryos obtained by crossing *Sprtn*^+/−^ and *Sprtn*^H/+^ mice. The H-line MEFs (H2, H3, H5 and H7) were obtained from E13.5 embryos produced by crossing *Sprtn*^+/−^ and *Sprtn*^F/+^ mice and immortalized by serial passage. For Cre-ER^T2^ expression in the H-line MEFs, cells were infected with retroviral vectors and selected with 3 μg ml^−1^ puromycin. The K-line MEFs (K3) were isolated from E13.5 embryos produced by crossing *Sprtn*^F/+^; *Cre-ER*^*T2*^ and *Sprtn*^F/+^ mice and immortalized by retroviral expression of SV40 T-antigen^44^. To induce conversion of the floxed allele to the KO allele in MEFs, cells were treated with methanol (MeOH) or 2 μM 4-OHT dissolved in MeOH for 2 days. For cell proliferation assays, 1.5 × 10^5^ cells were seeded in 10 cm dishes and cell numbers were recorded every 24 h for 4 days.

*Sprtn*^+/+^ and *Sprtn*^H/H^ primary lung fibroblasts were isolated from lung tissues of *Sprtn*^+/+^ and *Sprtn*^H/H^ male mice, respectively. Briefly, the lungs were chopped into small pieces and allowed to sit for few minutes in 100 mm dishes. Culture medium (DMEM supplemented with 10% FBS, non-essential amino acids, sodium pyruvate and 2-mercaptoethanol) was added to the dishes and cells were cultured for 4 days. All experiments were performed within three to six passages.

Prostate epithelial cells were isolated as described previously with modifications^45^. Briefly, prostate tissues were chopped into small pieces, minced in 1 ml of phosphate-buffered saline (PBS) containing 0.5% BSA and 2 mM EDTA using gentleMACS Dissociator (Miltenyi Biotec) and incubated with 7 mg ml^−1^ Liberase blendzyme 3 (Roche) at 37 °C for 30 min. After subsequent dissociation with gentleMACS Dissociator and centrifugation (1,000 r.p.m., 15 s), cell suspensions were collected and cleared from undigested tissues using a cell strainer. Prostate epithelial cells were then co-cultured with irradiated (3,000 rad) J2 fibroblast feeder cells^46^ in DMEM/F12 (3:1) supplemented with 10% FBS, 25 ng ml^−1^ hydrocortisone, 0.125 ng ml^−1^ EGF, 5 μg ml^−1^ insulin, 0.1 nM cholera toxin and 250 ng ml^−1^ Fungizone. Experiments were performed using epithelial cells at passage 4 after separation from feeder cells.

### Flow cytometry

For analyses of DNA content, cells were harvested and fixed with 70% ethanol at −20 °C overnight. Fixed cells were stained with propidium iodide (PI) solution (50 μg ml^−1^ PI, 10 μg ml^−1^ RNase A, 0.05% Nonidet P-40 (NP-40)) for at least 30 min and analysed by FACS Canto II (BD Biosciences). Percentages of the cell cycle phases were estimated using ModFit LT (Verity Software House). For detection of apoptosis, adherent and floating cells were harvested and stained with Annexin V-APC (BD Biosciences) for 15 min at room temperature. Cells were then stained with 100 μg ml^−1^ PI and analysed by the flow cytometer.

To analyze S-phase progression, cells were pulse labelled with 10 μM BrdU for 30 min and chased in BrdU-free media every 2 h. Cells were harvested and fixed in 70% ethanol at −20 °C overnight. DNA was denatured by suspending cells in 2 N HCl with 0.5% Triton X-100 for 30 min at room temperature. After neutralization with 0.1 M sodium borate (pH 8.5), cells were labelled with 1 μg of fluorescein isothiocyanate-conjugated anti-BrdU antibody (Roche, #11202693001) for 30 min at room temperature. Finally, cells were stained with PI solution (50 μg ml^−1^ PI, 10 μg ml^−1^ RNase A, 0.05% NP-40) for at least 30 min and analysed by flow cytometry. For phospho-histone H3 (Ser10), cells were incubated with 40 ng ml^−1^ nocodazole (Sigma) for 4 h, harvested and fixed with 70% ethanol at −20 °C overnight. Cells were washed once with PBS containing 1% BSA (1% BSA/PBS) and permeabilized with 1% BSA/PBS containing 0.25% Triton X-100 for 5 min at 4 °C. After washing with 1% BSA/PBS, cells were resuspended in 1 μg of anti-phospho-H3 (Millipore, #06-570) in 1% BSA/PBS containing 10% normal goat serum and incubated overnight at 4 °C. Cells were washed once with 1% BSA/PBS and incubated with 7 μg of Alexa Fluor 488-labelled goat anti-rabbit IgG (Invitrogen, #A11034) in 1% BSA/PBS containing 10% normal goat serum for 1 h at 4 °C. After washing with 1% BSA/PBS, cells were incubated in PI solution for at least 30 min and analysed by flow cytometry.

### Cytogenetics

Mitotic cells were enriched by colcemid treatment for 2 h and harvested by tapping plates. Cells were then swollen in hypotonic buffer for 25 min, fixed and dropped on glass slides. Slides were baked at 90 °C for 90 min and stained with Leishman’s stain solution for 1–3 min. After a brief rinse with running water, slides were dried at room temperature and analysed using a bright-field microscope. A metaphase spread was considered positive for breakage if any minor anomalies, major anomalies or radial configurations were observed.

### Chromatin fractionation and western blot

Cells were lysed in NETN lysis buffer (20 mM Tris–HCl, pH 8.0, 100 mM NaCl, 0.5% NP-40, 1 mM EDTA, 0.5 mM NaF, 1 mM β-glycerophosphate) supplemented with protease inhibitor mix (Sigma) followed by sonication. Chromatin-enriched fractionations were isolated by a previously described method^47^ with modifications. Briefly, cells were incubated in buffer A (10 mM Pipes (pH 6.8), 100 mM NaCl, 3 mM MgCl_2_, 300 mM sucrose, 1 mM EGTA, 0.2% Triton X-100, protease inhibitor mix (Sigma)) at 4 °C with rotation for 5 min. After centrifugation, pellets were washed again with buffer A and then incubated on ice for 30 min in buffer B (50 mM Tris-HCl (pH 7.5), 150 mM NaCl, 0.1% SDS) containing 500 U ml^−1^ Benzonase (Merck Millipore) followed by sonication. Supernatants containing solubilized chromatin proteins were recovered after centrifugation (14,000 r.p.m., 10 min). Thirty micrograms of proteins was separated by SDS–PAGE, transferred to nitrocellulose membranes and probed with antibodies. Antibodies against phospho-Chk1 (Ser345) (#2348, 1:1,000) and histone H3 (#9715, 1:5,000) were purchased from Cell Signaling Technology. Anti-Chk1 (#sc-8408, 1:1,000), anti-PCNA (#sc-56, 1:2,000) and anti-MCM7 (#sc-9966, 1:1,000) antibodies were from Santa Cruz Biotechnology. Anti-Chk2 antibodies were obtained from BD Biosciences (#611570, 1:1,000). Anti-Flag (#F1804, 1:2,000) and anti-β-actin (#A5316, 1:5,000) antibodies were purchased from Sigma. Mouse anti-human Spartan antibodies were reported previously^11^.

### Immunofluorescence and microscopy

Cells were fixed with 4% paraformaldehyde for 10 min at room temperature and permeabilized for 10 min with 0.2% Triton X-100. Cells were blocked for 1 h in PBS containing 3% BSA (Rad51), 1% normal goat serum (53BP1, γH2AX) or 3% normal goat serum (γH2AX for primary lung fibroblasts) and incubated with primary antibodies diluted in the respective blocking solution for 1 h at room temperature. Antibodies used were: rabbit anti-Rad51 (Calbiochem, #PC130, 1:100), rabbit anti-53BP1 (a gift from Zhenkun Lou, 1:500) and rabbit anti-γH2AX (Cell Signaling Technology, #2577, 1:500). After washing with PBS, cells were stained with secondary antibodies (Invitrogen, Alexa fluor 488 goat anti-rabbit IgG, #A11034, 1:2,000) diluted in the blocking solution for 30 min at room temperature. Cells were washed with PBS, stained with DAPI (0.1 μg ml^−1^, Sigma) for 5 min and mounted with ProLong Gold (Invitrogen). For analyses of micronuclei and chromatin bridges, cells were fixed as above or with methanol:acetone (3:1) for 10 min at −20 °C and permeabilized for 10 min with 0.2% Triton X-100 at room temperature, stained with DAPI (0.1 μg ml^−1^, Sigma) for 5 min and mounted with ProLong Gold (Invitrogen). Slides were viewed and photographed on a Zeiss fluorescent microscope with a × 100 objective.

### DNA fiber assays

Cells were labelled with 25 μM IdU (Sigma) for 15 min, washed twice with pre-warmed PBS and labelled with 200 μM CldU (Sigma) for 15 min. To measure fork progression at DNA lesions, cells were irradiated with 40 J m^−2^ ultraviolet in 5 ml media containing 1% FBS in 100 mm dish between IdU and CldU labelling. Labelled cells were harvested and mixed with unlabelled cells at 1:40 and concentrations were adjusted to 3–5 × 10^5^ ml^−1^ in PBS. Five microlitres of cell suspension was dropped on a glass slide and cells were lysed by adding 15 μl spreading buffer (200 mM Tris–HCl, pH 7.4, 50 mM EDTA, 0.5% SDS). After 8 min incubation at room temperature, DNA was spread by tilting glass slides and fixed with methanol/acetic acid (3:1) for 10 min. Slides were incubated with 2.5 M HCl for 1 h, neutralized by rinsing twice with 0.1 M sodium borate (pH 8.5), washed with PBS, blocked with PBS containing 5% goat serum for 1 h and incubated with anti-IdU antibodies (BD Biosciences, #347580, 1:100 in PBS containing 5% goat serum) overnight at 4 °C. After washing with low-salt TBST (36 mM Tris–HCl, pH 8.0, 50 mM NaCl, 0.5% Tween-20) for 20 min and then twice with PBST (PBS containing 0.2% Tween-20), slides were incubated with anti-CldU antibodies (Abcam, #ab6326, 1:1,000 in PBS containing 5% goat serum) overnight at 4 °C. Slides were washed three times with PBST and incubated with secondary antibodies (Invitrogen, Alexa fluor 594 anti-mouse IgG, #A11032, Alexa fluor 488 anti-rat IgG, #A11006, 1:2,000 in PBS containing 5% goat serum) for 30 min at 37 °C. After washing with PBST, slides were mounted with ProLong Gold (Invitrogen). Slides were viewed and photographed on a Zeiss fluorescent microscope with a × 100 objective. The lengths of IdU and CldU tracts were measured using the ImageJ software.

### Quantitative reverse transcription-PCR

Total RNA was isolated from the kidneys and lungs of *Sprtn*^+/+^ and *Sprtn*^H/H^ mice using TRIzol (Invitrogen) and from MEFs and IAT using RNeasy Kit (Qiagen). cDNAs were generated using oligo(dT) primers and SuperScript III (Invitrogen). Quantitative PCR was performed in triplicate for each sample on CFX96 (Bio-Rad) using iTaq Universal SYBR Green Supermix (Bio-Rad) or on ABI PRISM 7900 Sequence Detection System (Applied Biosystems) using SYBR green PCR Master Mix (Applied Biosystems). To quantify the wild-type *Sprtn* transcripts, primers were designed at the junction of exon 2 and exon 3 (forward primer: 5′- GGACCTTGTAGAGACTCTTTTG -3′) and in exon 4 (reverse primer: 5′- CCTCATCATGGAAAGTGTGG -3′) (Supplementary Fig. 4a). *Gapdh* was amplified using forward primer (5′- AGAACATCATCCCTGCATCC -3′) and reverse primer (5′- CACATTGGGGGTAGGAACAC -3′) and used for normalization between samples. For senescence-associated markers, following primers were used: *p16*^*Ink4a*^ forward, 5′- CCCAACGCCCCGAACT -3′; *p16*^*Ink4a*^ reverse, 5′- GCAGAAGAGCTGCTACGTGAA -3′; *p19*^*ARF*^ forward, 5′- AACTCTTTCGGTCGTACCCC -3′; *p19*^*ARF*^ reverse, 5′- GCGTGCTTGAGCTGAAGCTA -3′; *Pai1* forward, 5′- TCAGAGCAACAAGTTCAACTACACTGAG -3′; *Pai1* reverse 5′- CCCACTGTCAAGGCTCCATCACTTGCCCCA -3′; *Il6* forward, 5′- GACAACTTTGGCATTGTGG -3′; *Il6* reverse, 5′- ATGCAGGGATGATGTTCTG -3′; *Gapdh* forward, 5′- ACCACAGTCCATGCCATCAC -3′; *Gapdh* reverse, 5′- TCCACCACCCTGTTGCTGTA -3′.

## Additional information

**How to cite this article:** Maskey, R. S. *et al*. Spartan deficiency causes genomic instability and progeroid phenotypes. *Nat. Commun.* 5:5744 doi: 10.1038/ncomms6744 (2014).

## Supplementary Material

Supplementary Information Supplementary Figures 1-7 and Supplementary Table 1

## Figures and Tables

**Table 1:** Genotypes of offspring and embryos from *Sprtn* ^+/−^ intercrosses.

**Developmental stages**	*** Sprtn *** ^**+/+**^	*** Sprtn *** ^**+/**−^	*** Sprtn *** ^−**/**−^	**Total**
E3.5	6 (24.0%)	13 (52.0%)	6 (24.0%)	25
E7.5	5 (25.0%)	15 (75.0%)	0	20
E9.5	5 (26.3%)	14 (73.7%)	0	19
E11.5	7 (31.8%)	15 (68.2%)	0	22
E13.5	6 (30.0%)	14 (70.0%)	0	20
P21	85 (41.5%)	120 (58.5%)	0	205

E, embryonic day; P, postnatal day.

**Table 2:** *In vitro* development of blastocysts.

**Genotype**	**Number of blastocysts**	**Number of blastocysts presenting**	
		**Zona hatching at day 3**	**ICM outgrowth at day 6**
* Sprtn * ^+/+^	6	6	6
* Sprtn * ^+/−^	13	12	12
* Sprtn * ^−/−^	6	0	0

ICM, inner cell mass.

**Table 3:** Karyotypes of primary lung fibroblasts.

**Lung fibroblast genotype (** ***n*** **)**	**Mitotic cells inspected**	**% Aneuploid figures**	**Karyotypes with the indicated chromosome number**						
			**37**	**38**	**39**	**40**	**41**	**42**	**43**
*Sprtn*^+/+^ (2)	50	12	0	3	1	44	1	1	0
*Sprtn*^H/H^ (2)	50	36	1	5	2	32	6	2	2

Karyotyping was performed with lung fibroblasts isolated from two mice per genotype (#45 and #46 for *Sprtn*^+/+^, #84 and #87 for *Sprtn*^H/H^) at passage 5. Twenty-five mitotic figures were inspected for each sample.

## References

[b1] Zeman M. K. & Cimprich K. A. Causes and consequences of replication stress. Nat. Cell Biol. 16, 2–9 (2014). 24366029 10.1038/ncb2897 PMC4354890

[b2] Negrini S., Gorgoulis V. G. & Halazonetis T. D. Genomic instability-an evolving hallmark of cancer. Nat. Rev. Mol. Cell Biol. 11, 220–228 (2010). 20177397 10.1038/nrm2858

[b3] Vijg J. & Suh Y. Genome instability and aging. Annu. Rev. Physiol. 75, 645–668 (2013). 23398157 10.1146/annurev-physiol-030212-183715

[b4] Flach J. . Replication stress is a potent driver of functional decline in ageing haematopoietic stem cells. Nature 512, 198–202 (2014). 25079315 10.1038/nature13619 PMC4456040

[b5] Murga M. . A mouse model of ATR-Seckel shows embryonic replicative stress and accelerated aging. Nat. Genet. 41, 891–898 (2009). 19620979 10.1038/ng.420 PMC2902278

[b6] Ruzankina Y. . Deletion of the developmentally essential gene ATR in adult mice leads to age-related phenotypes and stem cell loss. Cell Stem Cell 1, 113–126 (2007). 18371340 10.1016/j.stem.2007.03.002 PMC2920603

[b7] Chang D. J. & Cimprich K. A. DNA damage tolerance: when it's OK to make mistakes. Nat. Chem. Biol. 5, 82–90 (2009). 19148176 10.1038/nchembio.139 PMC2663399

[b8] Sale J. E., Lehmann A. R. & Woodgate R. Y-family DNA polymerases and their role in tolerance of cellular DNA damage. Nat. Rev. Mol. Cell Biol. 13, 141–152 (2012). 22358330 10.1038/nrm3289 PMC3630503

[b9] Centore R. C., Yazinski S. A., Tse A. & Zou L. Spartan/C1orf124, a reader of PCNA ubiquitylation and a regulator of UV-induced DNA damage response. Mol. Cell 46, 625–635 (2012). 22681887 10.1016/j.molcel.2012.05.020 PMC3575090

[b10] Ghosal G., Leung J. W., Nair B. C., Fong K. W. & Chen J. Proliferating cell nuclear antigen (PCNA)-binding protein C1orf124 is a regulator of translesion synthesis. J. Biol. Chem. 287, 34225–34233 (2012). 22902628 10.1074/jbc.M112.400135 PMC3464530

[b11] Machida Y., Kim M. S. & Machida Y. J. Spartan/C1orf124 is important to prevent UV-induced mutagenesis. Cell Cycle 11, 3395–3402 (2012). 22894931 10.4161/cc.21694 PMC3466550

[b12] Davis E. J. . DVC1 (C1orf124) recruits the p97 protein segregase to sites of DNA damage. Nat. Struct. Mol. Biol. 19, 1093–1100 (2012). 23042607 10.1038/nsmb.2394

[b13] Mosbech A. . DVC1 (C1orf124) is a DNA damage-targeting p97 adaptor that promotes ubiquitin-dependent responses to replication blocks. Nat. Struct. Mol. Biol. 19, 1084–1092 (2012). 23042605 10.1038/nsmb.2395

[b14] Juhasz S. . Characterization of human Spartan/C1orf124, an ubiquitin-PCNA interacting regulator of DNA damage tolerance. Nucleic Acids Res. 40, 10795–10808 (2012). 22987070 10.1093/nar/gks850 PMC3510514

[b15] Kim M. S. . Regulation of error-prone translesion synthesis by Spartan/C1orf124. Nucleic Acids Res. 41, 1661–1668 (2013). 23254330 10.1093/nar/gks1267 PMC3561950

[b16] Hendzel M. J. . Mitosis-specific phosphorylation of histone H3 initiates primarily within pericentromeric heterochromatin during G2 and spreads in an ordered fashion coincident with mitotic chromosome condensation. Chromosoma 106, 348–360 (1997). 9362543 10.1007/s004120050256

[b17] Juan G. . Histone H3 phosphorylation and expression of cyclins A and B1 measured in individual cells during their progression through G2 and mitosis. Cytometry 32, 71–77 (1998). 9627219 10.1002/(sici)1097-0320(19980601)32:2<71::aid-cyto1>3.0.co;2-h

[b18] Rogakou E. P., Pilch D. R., Orr A. H., Ivanova V. S. & Bonner W. M. DNA double-stranded breaks induce histone H2AX phosphorylation on serine 139. J. Biol. Chem. 273, 5858–5868 (1998). 9488723 10.1074/jbc.273.10.5858

[b19] Fenech M. . Molecular mechanisms of micronucleus, nucleoplasmic bridge and nuclear bud formation in mammalian and human cells. Mutagenesis 26, 125–132 (2011). 21164193 10.1093/mutage/geq052

[b20] Harrigan J. A. . Replication stress induces 53BP1-containing OPT domains in G1 cells. J. Cell Biol. 193, 97–108 (2011). 21444690 10.1083/jcb.201011083 PMC3082192

[b21] Lukas C. . 53BP1 nuclear bodies form around DNA lesions generated by mitotic transmission of chromosomes under replication stress. Nat. Cell Biol. 13, 243–253 (2011). 21317883 10.1038/ncb2201

[b22] Dawlaty M. M. & van Deursen J. M. Gene targeting methods for studying nuclear transport factors in mice. Methods 39, 370–378 (2006). 16887365 10.1016/j.ymeth.2006.06.009

[b23] Baker D. J. . BubR1 insufficiency causes early onset of aging-associated phenotypes and infertility in mice. Nat. Genet. 36, 744–749 (2004). 15208629 10.1038/ng1382

[b24] Baker D. J. . Early aging-associated phenotypes in Bub3/Rae1 haploinsufficient mice. J. Cell Biol. 172, 529–540 (2006). 16476774 10.1083/jcb.200507081 PMC2063673

[b25] Hasty P., Campisi J., Hoeijmakers J., van Steeg H. & Vijg J. Aging and genome maintenance: lessons from the mouse? Science 299, 1355–1359 (2003). 12610296 10.1126/science.1079161

[b26] Baker D. J. . Opposing roles for p16Ink4a and p19Arf in senescence and ageing caused by BubR1 insufficiency. Nat. Cell Biol. 10, 825–836 (2008). 18516091 10.1038/ncb1744 PMC2594014

[b27] Hendel A. . PCNA ubiquitination is important, but not essential for translesion DNA synthesis in mammalian cells. PLoS Genet. 7, e1002262 (2011). 21931560 10.1371/journal.pgen.1002262 PMC3169526

[b28] Bemark M., Khamlichi A. A., Davies S. L. & Neuberger M. S. Disruption of mouse polymerase zeta (Rev3) leads to embryonic lethality and impairs blastocyst development in vitro. Curr. Biol. 10, 1213–1216 (2000). 11050391 10.1016/s0960-9822(00)00724-7

[b29] Esposito G. . Disruption of the Rev3l-encoded catalytic subunit of polymerase zeta in mice results in early embryonic lethality. Curr. Biol. 10, 1221–1224 (2000). 11050393 10.1016/s0960-9822(00)00726-0

[b30] Wittschieben J. . Disruption of the developmentally regulated Rev3l gene causes embryonic lethality. Curr. Biol. 10, 1217–1220 (2000). 11050392 10.1016/s0960-9822(00)00725-9

[b31] Kajiwara K. . Sez4 gene encoding an elongation subunit of DNA polymerase zeta is required for normal embryogenesis. Genes Cells 6, 99–106 (2001). 11260255 10.1046/j.1365-2443.2001.00410.x

[b32] Van Sloun P. P. . Involvement of mouse Rev3 in tolerance of endogenous and exogenous DNA damage. Mol. Cell. Biol. 22, 2159–2169 (2002). 11884603 10.1128/MCB.22.7.2159-2169.2002 PMC133679

[b33] Lange S. S., Wittschieben J. P. & Wood R. D. DNA polymerase zeta is required for proliferation of normal mammalian cells. Nucleic Acids Res. 40, 4473–4482 (2012). 22319213 10.1093/nar/gks054 PMC3378892

[b34] Bergoglio V. . DNA synthesis by Pol eta promotes fragile site stability by preventing under-replicated DNA in mitosis. J. Cell Biol. 201, 395–408 (2013). 23609533 10.1083/jcb.201207066 PMC3639397

[b35] Betous R. . Role of TLS DNA polymerases eta and kappa in processing naturally occurring structured DNA in human cells. Mol. Carcinog. 48, 369–378 (2009). 19117014 10.1002/mc.20509 PMC2696892

[b36] Rey L. . Human DNA polymerase eta is required for common fragile site stability during unperturbed DNA replication. Mol. Cell. Biol. 29, 3344–3354 (2009). 19380493 10.1128/MCB.00115-09 PMC2698728

[b37] Walsh E., Wang X., Lee M. Y. & Eckert K. A. Mechanism of replicative DNA polymerase delta pausing and a potential role for DNA polymerase kappa in common fragile site replication. J. Mol. Biol. 425, 232–243 (2013). 23174185 10.1016/j.jmb.2012.11.016 PMC3540124

[b38] Lopez-Otin C., Blasco M. A., Partridge L., Serrano M. & Kroemer G. The hallmarks of aging. Cell 153, 1194–1217 (2013). 23746838 10.1016/j.cell.2013.05.039 PMC3836174

[b39] Garinis G. A., van der Horst G. T., Vijg J. & Hoeijmakers J. H. DNA damage and ageing: new-age ideas for an age-old problem. Nat. Cell Biol. 10, 1241–1247 (2008). 18978832 10.1038/ncb1108-1241 PMC4351702

[b40] Lessel D. . Mutations in SPRTN cause early onset hepatocellular carcinoma, genomic instability and progeroid features. Nat. Genet. 46, 1239–1244 (2014). 25261934 10.1038/ng.3103 PMC4343211

[b41] Ventura A. . Restoration of p53 function leads to tumour regression *in vivo*. Nature 445, 661–665 (2007). 17251932 10.1038/nature05541

[b42] van Deursen J., Boer J., Kasper L. & Grosveld G. G2 arrest and impaired nucleocytoplasmic transport in mouse embryos lacking the proto-oncogene CAN/Nup214. EMBO J. 15, 5574–5583 (1996). 8896451 PMC452302

[b43] Baker D. J. . Clearance of p16Ink4a-positive senescent cells delays ageing-associated disorders. Nature 479, 232–236 (2011). 22048312 10.1038/nature10600 PMC3468323

[b44] Hahn W. C. . Enumeration of the simian virus 40 early region elements necessary for human cell transformation. Mol. Cell. Biol. 22, 2111–2123 (2002). 11884599 10.1128/MCB.22.7.2111-2123.2002 PMC133688

[b45] Barclay W. W. & Cramer S. D. Culture of mouse prostatic epithelial cells from genetically engineered mice. Prostate 63, 291–298 (2005). 15599944 10.1002/pros.20193

[b46] Liu X. . ROCK inhibitor and feeder cells induce the conditional reprogramming of epithelial cells. Am. J. Pathol. 180, 599–607 (2012). 22189618 10.1016/j.ajpath.2011.10.036 PMC3349876

[b47] Kannouche P. L., Wing J. & Lehmann A. R. Interaction of human DNA polymerase eta with monoubiquitinated PCNA: a possible mechanism for the polymerase switch in response to DNA damage. Mol. Cell 14, 491–500 (2004). 15149598 10.1016/s1097-2765(04)00259-x

