# Supplementary material for: Spartan deficiency causes genomic instability and progeroid phenotypes
Source: Nat Commun. 2014 Dec 11;5:5744. doi: 10.1038/ncomms6744 (PMC4269170; doi:10.1038/ncomms6744)
Supplement: Supplementary Information — Supplementary Figures 1-7 and Supplementary Table 1 [file ncomms6744-s1.pdf]

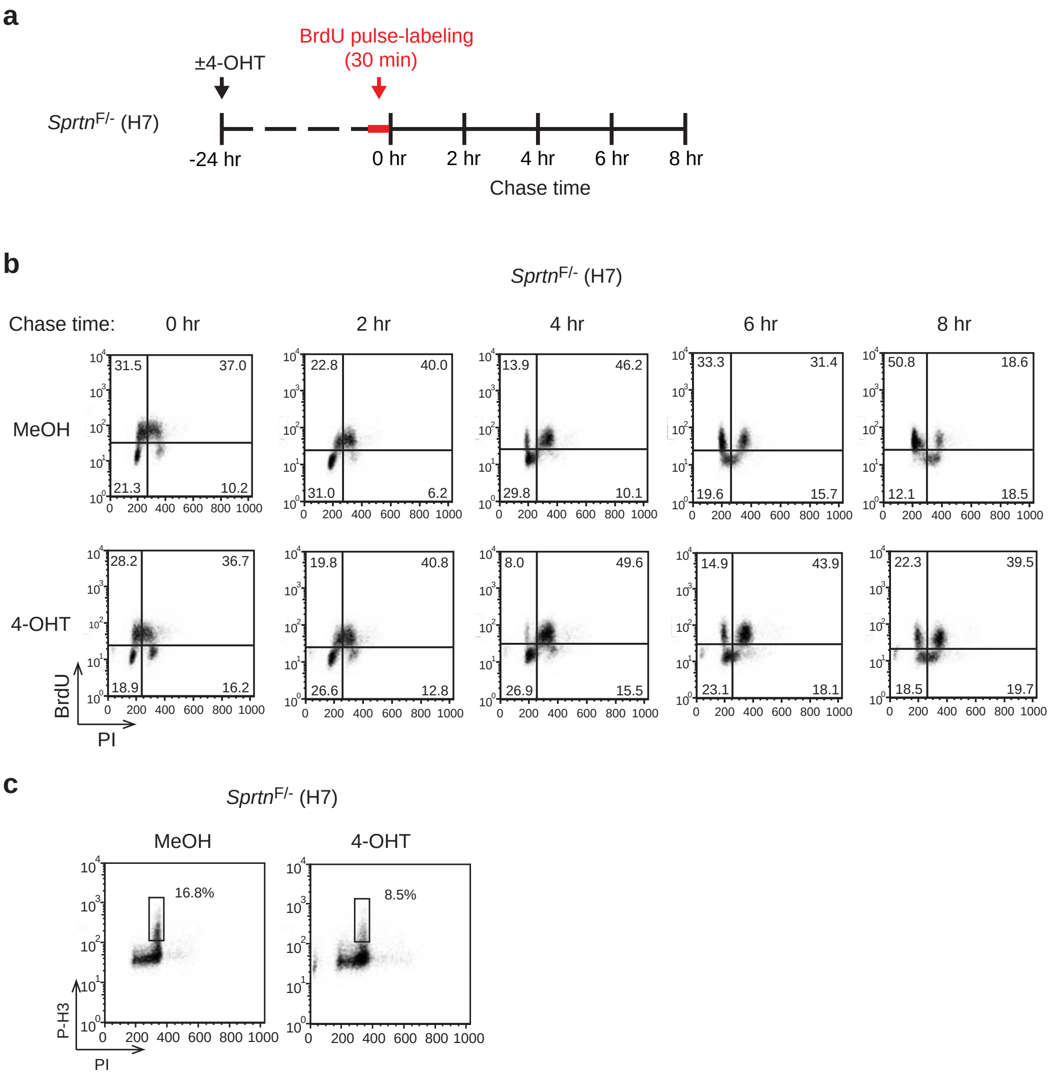

Supplementary Figure 1 Cell-cycle analyses of *Sprtn*$^{-/-}$ MEFs. (**a**) Schematic representation of BrdU pulse-chase experiments. *Sprtn*$^{F/-}$ MEFs treated with MeOH or 4-OHT for 24 hr were pulse-labeled with 10 µM BrdU for 30 min. BrdU-labeled S-phase cells were then chased in BrdU-free media, harvested at the indicated time points (every 2 hr) and analyzed for BrdU and PI staining by flow cytometry. (**b**) Analyses of S-phase progression by BrdU pulse-chase experiments in *Sprtn*$^{F/-}$ cells. Percentages of cells in each quadrant are indicated. (**c**) Analyses of phospho-histone H3 (mitotic marker) in *Sprtn*$^{F/-}$ MEFs. After 48 hr of MeOH or 4-OHT treatment, *Sprtn*$^{F/-}$ MEFs were incubated with 40 ng ml^{-1} Nocodazole for 4 hr and harvested. After staining with phospho-histone H3 (Ser10) antibodies and PI, cells were analyzed by flow cytometry. Boxes indicate phospho-histone H3 positive populations (mitotic populations).

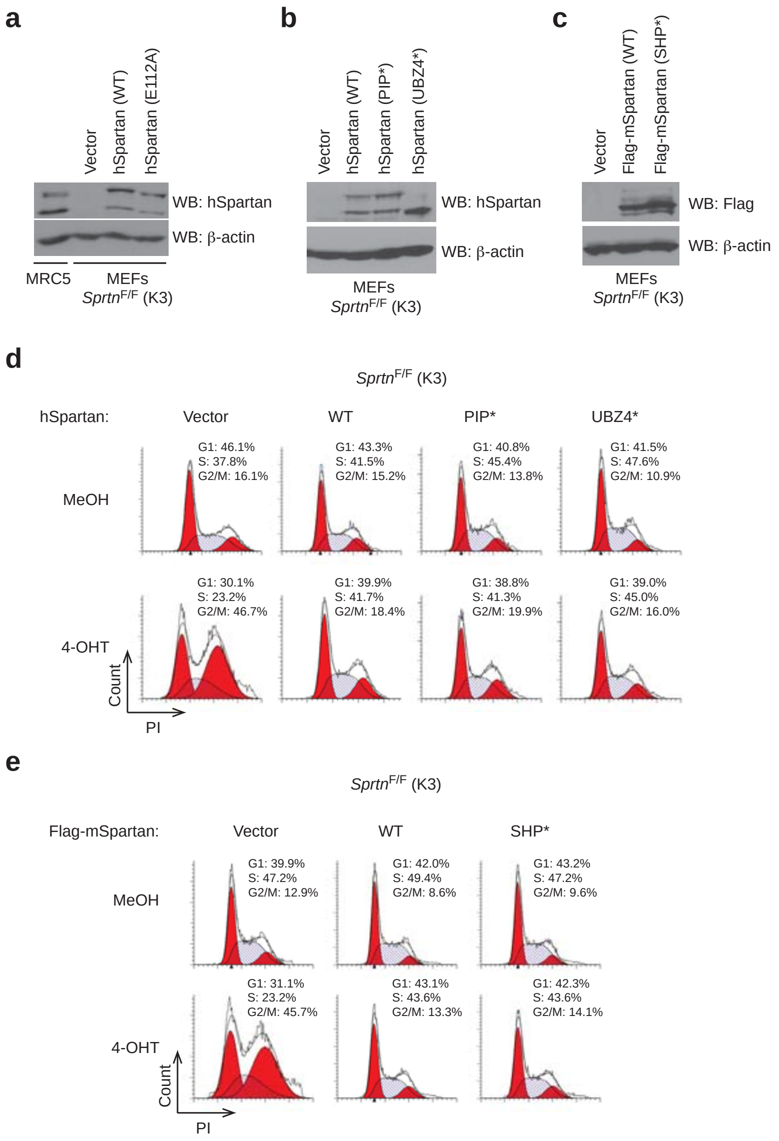

Supplementary Figure 2 Effects of mutations in the Spartan domains on the cell cycle. (**a**-**c**) Western blotting showing the expression of the indicated Spartan proteins in *Sprtn*$^{F/F}$; Cre-ER^{T2} MEFs (K3). hSpartan, human Spartan; mSpartan, mouse Spartan. Human fibroblast cell line MRC5 is shown as a positive control for the endogenous expression level of human Spartan. Spartan proteins are detected as two major bands due to its monoubiquitination. β-actin is shown as a loading control. (**d**,**e**) Cell-cycle profiling of *Sprtn*$^{F/F}$; Cre-ER^{T2} MEFs (K3) expressing the indicated Spartan proteins. Cells were treated with MeOH or 4-OHT for 48 hr, stained with PI and analyzed by flow cytometry.

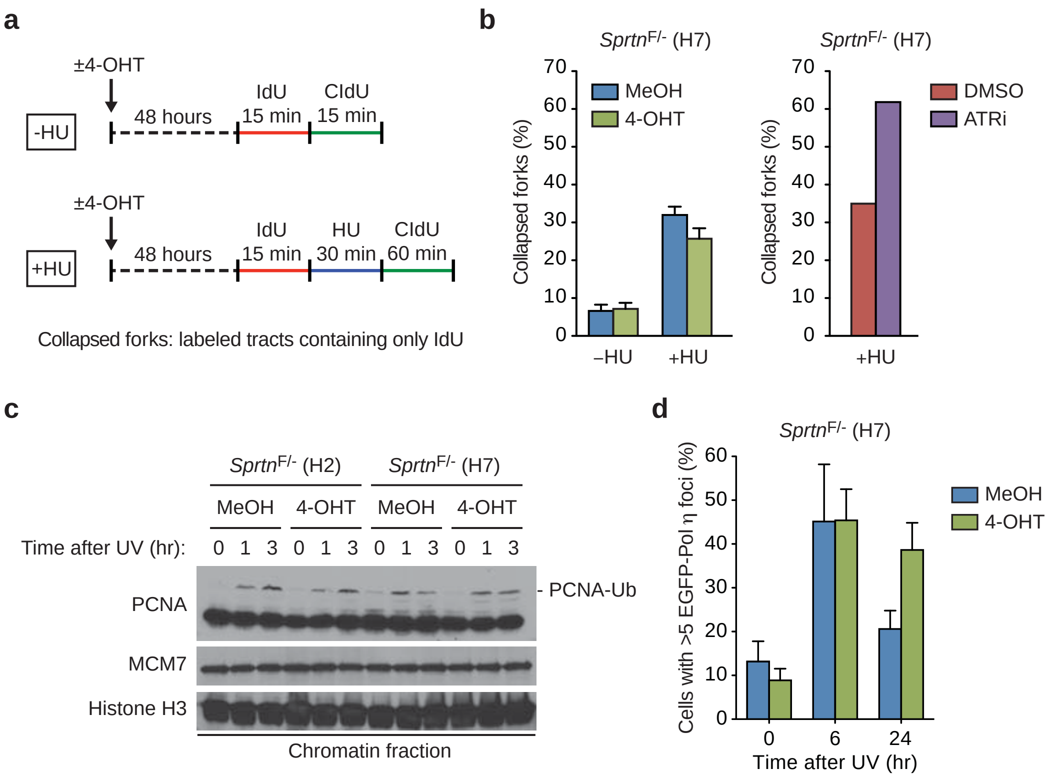

Supplementary Figure 3 Replication fork restart, UV-induced PCNA ubiquitination and Pol η focus formation in *Sprtn*$^{–/–}$ MEFs. (**a**) Schematic representation of DNA fiber assays used to measure collapsed forks (HU-induced stalled forks that failed to restart). *Sprtn*$^{F/–}$ MEFs treated with MeOH or 4-OHT for 48 hr were pulse-labeled with IdU for 15 min, treated with 5 mM HU for 30 min and released into CldU-containing media for 60 min. In the experiments without HU treatments, cells were sequentially labeled with IdU (15 min) and CldU (15 min). (**b**) Analyses of replication fork restart in *Sprtn*$^{–/–}$ MEFs. The left panel shows quantification of collapsed replication forks (labeled tracts containing only IdU) as percentage of all IdU-labeled tracts. Mean ± s.d. of three independent experiments is shown. The right panel shows a positive control for induced replication fork collapse, in which *Sprtn*$^{F/–}$ (H7) cells were incubated with 5 μM ATRi (ATR inhibitor, VE-821) 30 min prior to and during 30 min incubation with HU. For each sample, at least 650 fibers were scored. (**c**) Western blotting analyses of PCNA ubiquitination in *Sprtn*$^{–/–}$ MEFs in response to UV treatment. The indicated *Sprtn*$^{F/–}$ MEFs treated with MeOH or 4-OHT for 48 hr were irradiated with 40 J m^{-2} UV and harvested after 1 or 3 hr. Fractions enriched for chromatin-associated proteins were isolated and probed for PCNA and MCM7. The position of ubiquitinated PCNA is indicated on right. Histone H3 is shown as a loading control. (**d**) Quantitation of cells containing EGFP-Pol η foci. *Sprtn*$^{F/–}$ MEFs stably expressing EGFP-Pol η were treated with MeOH or 4-OHT for 30 hr, irradiated with 40 J m^{-2} UV and scored for EGFP-Pol η foci at the indicated time points. At least 200 cells were counted and percentages of cells containing more than 5 foci are shown. Experiments were performed in triplicate and mean ± s.d. is shown.

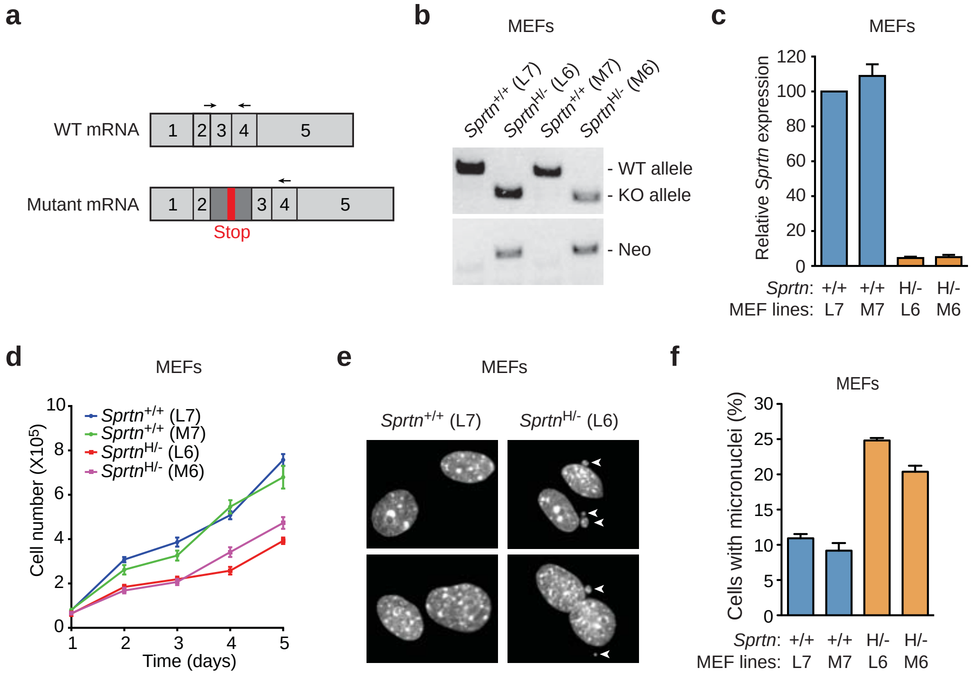

Supplementary Figure 4 ***Sprtn*$^{H/-}$ MEFs exhibit growth defects and genome instability.** (**a**) A scheme of wild-type and mutant *Sprtn* transcripts. The *Sprtn* hypomorphic allele produces wild-type and mutant mRNAs due to the cryptic exon in the Neo cassette. To quantitate only the wild-type mRNAs in *Sprtn* hypomorphic mice, the forward primer was designed at the junction of exon 2 and exon 3, and the reverse primer in exon 4. Positions of the primers are indicated by arrows. A cryptic exon from the *Neo* gene is shown between exon 2 and exon 3 of the mutant transcript. Red bar indicates a stop codon. (**b**) PCR-based genotyping of *Sprtn*$^{+/+}$ and *Sprtn*$^{H/-}$ MEFs. (**c**) qPCR analyses of *Sprtn* mRNA levels in *Sprtn*$^{+/+}$ and *Sprtn*$^{H/-}$ primary MEFs. Total RNA was isolated from the indicated MEF lines and *Sprtn* expression was measured three times by RT-PCR and mean ± s.d. is shown. Values were normalized to *Gapdh* and presented relative to *Sprtn*$^{+/+}$ (L7). (**d**) Proliferation of primary MEFs with the indicated genotypes at passage 3. Values are mean ± s.d. of three independent experiments. (**e**) Representative images of DAPI-stained nuclei of *Sprtn*$^{+/+}$ and *Sprtn*$^{H/-}$ primary MEFs. Arrowheads indicate micronuclei. (**f**) Quantitation of micronuclei-containing cells. At least 300 cells were scored for micronuclei in each experiment and percentages of positive cells are shown. Values are mean ± s.d. of three independent experiments. $p < 0.0001$ (H/- group versus +/+ group), two-tailed unpaired t-test.

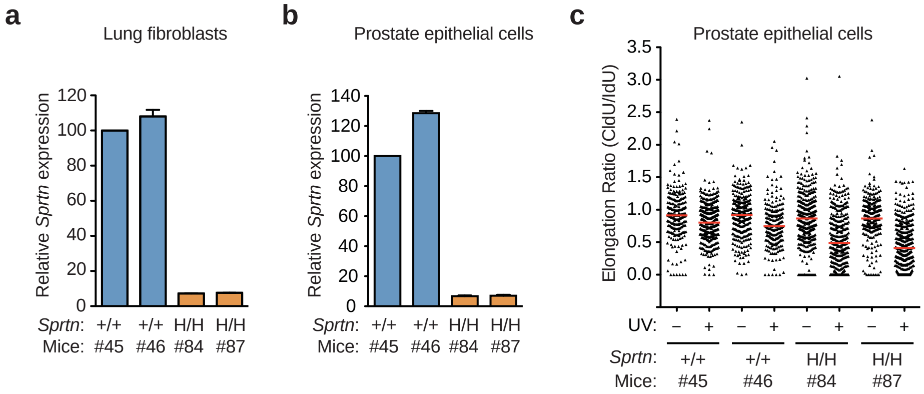

Supplementary Figure 5 ***Sprtn*$^{\mathbf{H/H}}$ primary cells exhibit UV-induced lesion bypass defects.** (**a**,**b**) qPCR analyses of *Sprtn* mRNA levels in *Sprtn*$^{+/+}$ and *Sprtn*$^{H/H}$ primary lung fibroblasts (**a**) and prostate epithelial cells (**b**) . Total RNA was isolated from the indicated cells and *Sprtn* expression was measured by RT-PCR. Values were normalized to *Gapdh* and shown relative to *Sprtn*$^{+/+}$ #45. Mean ± s.d. from three independent experiments is shown. (**c**) Effects of UV irradiation on replication forks. DNA fiber assays were performed with *Sprtn*$^{+/+}$ and *Sprtn*$^{H/H}$ primary prostate epithelial cells with or without UV irradiation (40 J m^{-2}) between IdU and CldU labeling. Distribution of replication forks at different CldU/IdU ratios is shown. A horizontal red line indicates median value. At least 200 fibers were scored for each sample.

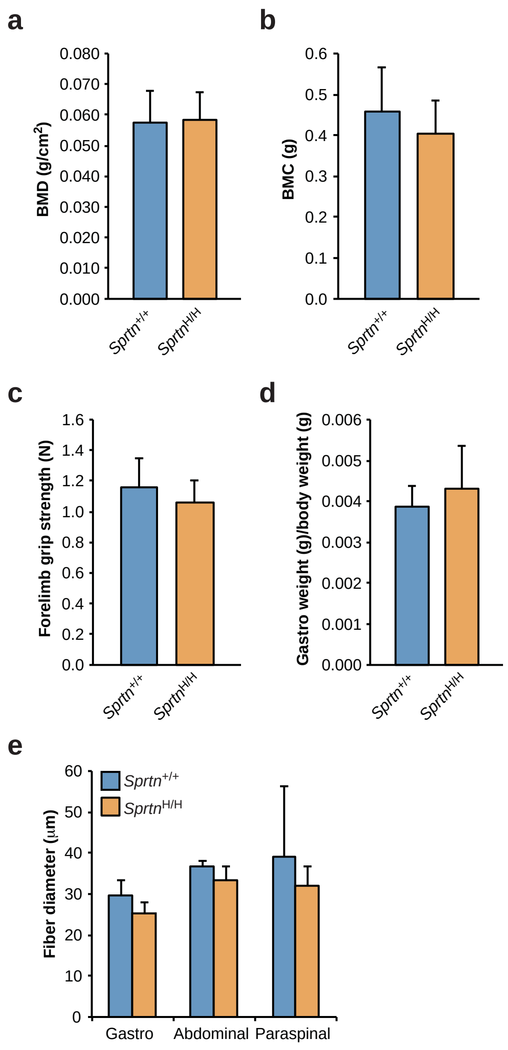

Supplementary Figure 6 Age-associated traits of *Sprtn* hypomorphic mice that are not accelerated with advancing age. (**a**,**b**) Using DEXA scanning, osteoporosis is not present in *Sprtn*$^{H/H}$ mice as bone mineral density (BMD) (**a**) and bone mineral content (BMC) (**b**) are normal. (**c**) Forelimb grip strength is normal in *Sprtn* hypomorphic mice. (**d**) Weight of gastrocnemius muscle relative to total body weight is unchanged with reduced levels of *Sprtn*. (**e**) Skeletal muscle fiber diameter measurements reveal no evidence for sarcopenia in 12-month-old female *Sprtn*$^{H/H}$ mice. Values presented in **a-e** represent mean ± s.d. (n = 5 *Sprtn*$^{+/+}$ and 5 *Sprtn*$^{H/H}$ mice in **a-d**, and n = 3 *Sprtn*$^{+/+}$ and 5 *Sprtn*$^{H/H}$ mice in **e**).

Supplementary Figure 7. Uncropped immunoblots

Figure 3c Raw Data:

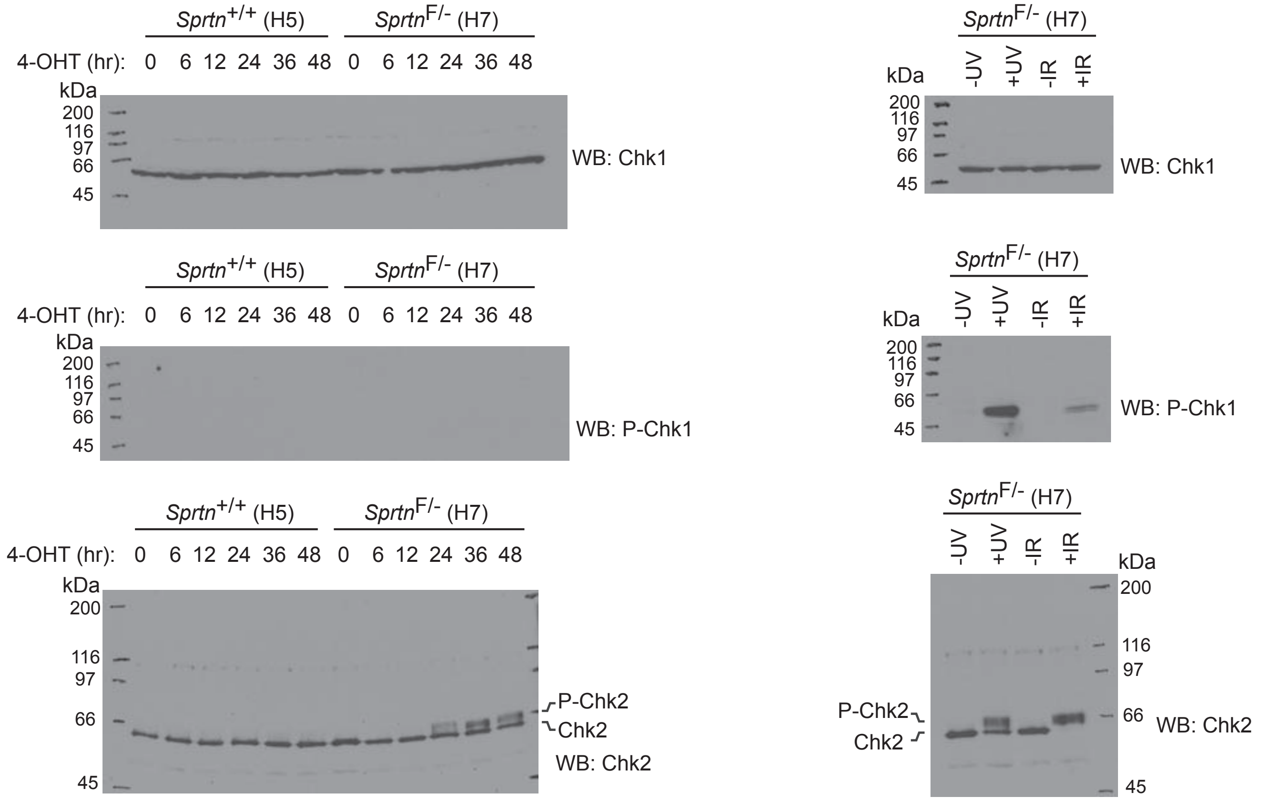

Supplementary Figure 2 Raw data:

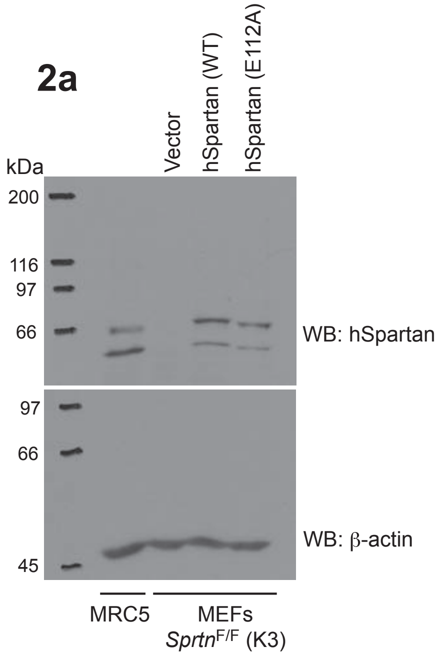

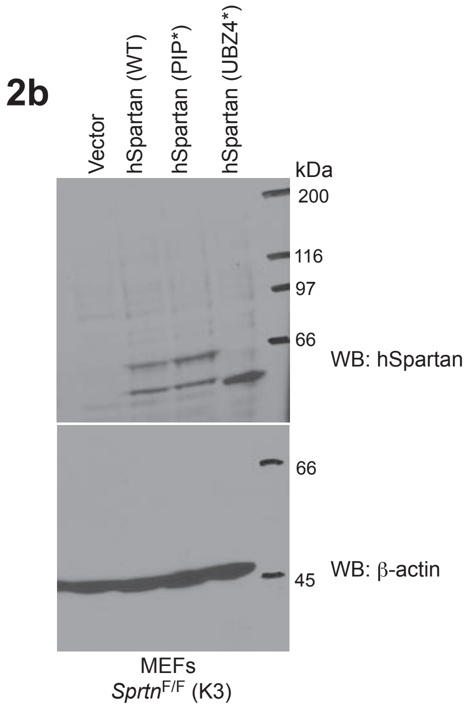

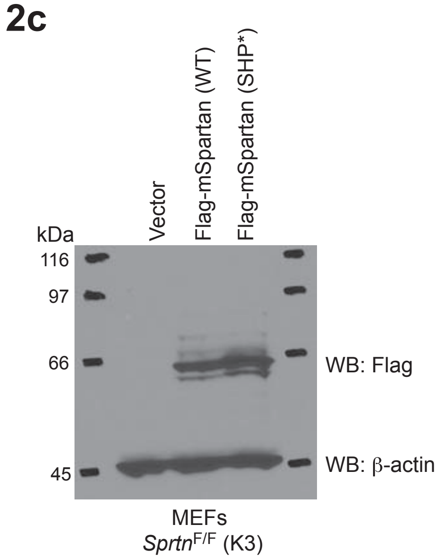

Supplementary Figure 3c Raw data

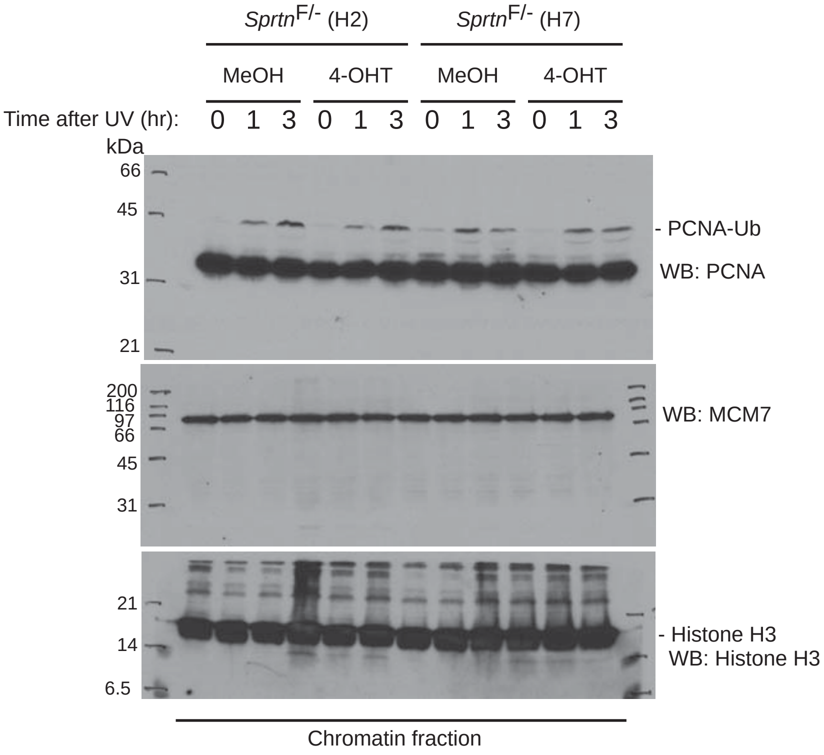

Supplementary Table 1. Karyotypes of primary MEFs.

MEF genotype (*n*)	Mitotic cells inspected	% aneuploid figures (s.d.)	Karyotypes with the indicated chromosome number						
			37	38	39	40	41	42	43
Sprtn$^{+/+}$ (4)	200	11 (3)	1	5	6	179	6	2	1
Sprtn$^{H/-}$ (4)	200	28 (5)	3	9	13	144	21	8	2

Karyotyping was performed with two independent cultures for two MEF lines per genotype (L7 and M7 for *Sprtn*$^{+/+}$, L6 and M6 for *Sprtn*$^{H/-}$) at passage 5. Fifty mitotic figures were inspected for each sample.
